# A Challenge for Contrastive L1/L2 Corpus Studies: Large Inter- and Intra-Individual Variation Across Morphological, but Not Global Syntactic Categories in Task-Based Corpus Data of a Homogeneous L1 German Group

**DOI:** 10.3389/fpsyg.2021.716485

**Published:** 2021-11-25

**Authors:** Anna Shadrova, Pia Linscheid, Julia Lukassek, Anke Lüdeling, Sarah Schneider

**Affiliations:** Department of German Studies and Linguistics, Humboldt-Universität zu Berlin, Berlin, Germany

**Keywords:** corpus linguistic analysis, quantitative linguistics, morphology, usage-based linguistics, verb morphology, noun morphology, language variation and corpus

## Abstract

In this paper, we present corpus data that questions the concept of native speaker homogeneity as it is presumed in many studies using native speakers (L1) as a control group for learner data (L2), especially in corpus contexts. Usage-based research on second and foreign language acquisition often investigates quantitative differences between learners, and usually a group of native speakers serves as a control group, but often without elaborating on differences within this group to the same extent. We examine inter-personal differences using data from two well-controlled German native speaker corpora collected as control groups in the context of second and foreign language research. Our results suggest that certain linguistic aspects vary to an extent in the native speaker data that undermines general statements about quantitative expectations in L1. However, we also find differences between phenomena: while morphological and syntactic sub-classes of verbs and nouns show great variability in their distribution in native speaker writing, other, coarser categories, like parts of speech, or types of syntactic dependencies, behave more predictably and homogeneously. Our results highlight the necessity of accounting for inter-individual variance in native speakers where L1 is used as a target ideal for L2. They also raise theoretical questions concerning a) explanations for the divergence between phenomena, b) the role of frequency distributions of morphosyntactic phenomena in usage-based linguistic frameworks, and c) the notion of the individual adult native speaker as a general representative of the target language in language acquisition studies or language in general.

## 1. Introduction

The frequency of occurrence of linguistic elements and categories, such as words, clause types, morphological, syntactic, or lexical features, has played a central role in usage-based linguistics (Ellis, 2002; Granger, 2005, 2015; Goldberg, 2006, 2013; Biber and Jones, 2009; Paquot and Granger, 2012; Zeldes, 2012; Bybee, 2013; Gries, 2013, 2014; Hirschmann et al., 2013; Bestgen and Granger, 2014; Gries and Ellis, 2015; Hirschmann, 2015; Diessel and Hilpert, 2016, among many others). In connectivist models of learning and acquisition, linguistic ability is modeled as the result of entrenchment of neuronal pathways through repeated exposure. Frequency is a crucial factor in deciding which combinations or connections emerge and persist (Croft, 2000; Tomasello, 2000, 2009; Bybee and Hopper, 2001; Goldberg et al., 2004; Schmitt, 2004; Gries and Wulff, 2005; Hoey, 2005; Ellis, 2006, 2012; Divjak and Caldwell-Harris, 2015; Ellis and Wulff, 2015, and many others). Since language learners are overall less exposed to target language input compared to native speakers, frequency has also served as an explanation for divergent degrees of language attainment in second language acquisition (SLA), for instance in studies that work with the concepts of over- and underuse (Paquot and Granger, 2012; Bestgen and Granger, 2014, and others), especially in connection with Contrastive Interlanguage Analysis, an influential method in learner corpus research (Granger, 2015). Deviations from native speaker frequencies found in corpora are frequently interpreted as evidence for true differences between native speakers and learners rather than random fluctuation. Typically, cumulative corpus counts or relative frequencies normalized to corpus size or a fixed number of tokens, such as one million words, are used for this.

This can be problematic, since native speakers are not monolithic in their use of language, as has been studied explicitly in variationist and socio-linguistic approaches (Eckert, 2016; Szmrecsanyi, 2017; Bayley, 2019), including SLA, e.g., Linford et al. (2016) and Gurzynski-Weiss et al. (2018). Careful analysis of inter-individual variability in L1 in quantitative learner corpus studies remains rare, as has been pointed out by Gries and Deshors (2014)^1^. This lack of attention can in part be attributed to limitations of the data as it tends to occur in corpora. Individual texts frequently contain only few instances of the categories of interest, especially where lexical or phraseological material is concerned (Shadrova, 2020, chap. 4), and thus often do not allow for a meaningful analysis of inter-individual variance. To a degree, this is unavoidable, since corpus data is not as neatly controllable with respect to the elicitation of linguistic features as some experimental data, and some features do not occur frequently unless prompted directly. Limited ability to consider inter- or intra-individual differences can also be due to corpus design, especially where data is collected without attribution to individuals (like web corpora) or texts in the data differ too much in text length, type, or genre to be easily comparable (like homework corpora collected over years). However, importantly, this common practice in quantitative corpus linguistics is also an extension of the underlying philosophy held by most models of language acquisition in usage-based linguistics—if frequency is modeled as somewhat stable in the in- and output, *there should be no problem* with cumulative data.

The data we present in this study suggests that this may not be true on all levels of granularity. Our two corpora of essays written by German native speakers, Falko (Reznicek et al., 2012) and Kobalt (Zinsmeister et al., 2012)^2^, were collected with the aim of maximally homogenizing the data regarding age, environment, and conditions of elicitation and prompt (intended to elicit homogeneous topic, register, and genre), as they were originally compiled as control group data in a contrastive L1/L2 paradigm^3^.

In spite of this maximally homogeneous composition, we find surprisingly high levels of inter-individual variation in the distribution of morphological categories of verbs and nouns and syntactic subclasses of verbs. At the same time, we find high convergence between participants regarding the distributions of global syntactic categories (parts of speech and syntactic dependencies). The purpose of this paper is to present and discuss these differences and similarities, and to highlight some of the repercussions of these findings on usage-based theory and corpus methodology. It is, to our best knowledge, the first corpus-based and quantitative account of both morphological and syntactic categories in homogeneous corpora of German. While we enter the discussion from a learner corpus perspective, we will not discuss learner data in this paper in order to give space for a discussion of what is designed as control group data. We argue that converging frequency distributions cannot be expected across levels of granularity even in socially and functionally highly homogeneous data. Rather, it appears that distributions converge on *some*, but not *all* linguistic levels. It follows that cumulative corpus accounts can be grossly misleading depending on the phenomenon they wish to investigate. They do not account for the full complexity of native speaker writing and may lead to over- or underestimations or incomplete models of true differences between L1 and L2.

The two stances—that cumulative data can be of sufficiently fine resolution and that native speakers can vary in their linguistic expression—are in principle not contradictory. Linguistic variationism, as we understand it, focuses on social, situational, or linguistic, but always *functional*, i.e., *stratified*, variability cf. Eckert (2016), Bayley (2019), and Szmrecsanyi (2019). This is expressed in a matrix of variants of a variable by factor, such as group membership (e.g., an individual's belonging to a certain age bracket, geographic area, cultural background, etc., cf. Dubois and Sankoff, 2001; Lüdeling, 2017; Szmrecsanyi, 2017); groups formed from less transparently available traits, such as aptitude, motivation; or functional variance, such as situational aspects, mode, genre, or register (Biber, 2012; Biber et al., 2016; Szmrecsanyi, 2019). More language-internal factors include stratified variance triggered by linguistic environments, such as the presence or absence of certain constructions of lexemes that may predict certain grammatical expressions, e.g., dative alternations or subject realization (Bernaisch et al., 2014; Deshors and Gries, 2016; Arroyo and Schulte, 2017; Cacoullos and Travis, 2019). Factors, whether they are language-internal or language-external, are mapped to predictable shifts in linguistic expression.

Needless to say, this perspective in the context of SLA research has fostered discussions around the necessity to redefine “the” native speaker, namely through “underscor[ing] the dangers of assuming what the target of L2 acquisition is” (Birdsong and Gertken, 2013, p.118). It has also raised attention to the question of how to carefully choose and specify what kind of group can legitimately serve as a control group for learner studies, for instance learners of other L2s, bilingual native speakers, instructors in a teaching setting, etc^4^.

This specification of the composition of the control group does not, however, constitute a break with the broad paradigm of L1/L2 comparison based on frequency of occurrence of linguistic elements—a comparison that only makes sense if a certain stability can be expected within a group or environment. The work we present here takes a closer look at differences that go beyond unanimous, clearly external factor-dependent shifts in a data set across speakers, highlighting linguistic expression at the level of individual text production and the challenges of its quantification. This is relevant since all text—whether we find it in large-scale, general corpora or in smaller, task-based corpora is the result of individual text production.

In the following sections, we will first give a short and necessarily broad introduction to the theoretical framework of some strands of usage-based linguistics as far as they concern language learning. We then briefly discuss previous research of individual differences in corpus linguistics and present the two corpora used in this study. Following this, we discuss our results and look into the role of priming as a possible explanatory concept for higher degrees of variation with the aim of highlighting the relevance of both inter- and intraindividual variation in L1. In the final section, we summarize the conclusions we draw from our observations for a) corpus methodology in general and b) theoretical aspects of usage-based linguistic frameworks in particular.

## 2. Literature Review

In this section, we will briefly introduce theoretical models as they touch aspects of our data analysis, and review previous literature into L1 variability in learner corpus research as well as priming as a procedural factor in language production. We will summarize the main points as they relate to our research question at the end of the section.

### 2.1. Native Speakers and the Concept of the Target Language

It is well-known that linguistic theory has long since been divided into more rationalist, Universal Grammar (UG)-based approaches vs. more empiricist, behaviorist approaches subsumed under the umbrella term of usage-based linguistics. This has abundant implications for the explanatory models including questions of learnability, the role of frequency (if any), the status of target language vs. native language, the relevance of input in language acquisition, as well as study design and the operationalization of concepts in both paradigms. We approach our data from a usage-based framework and will hence not discuss UG-based approaches here^5^.

As Ortega (2015b) points out, usage-based approaches do not constitute a single monolithic framework, but describe a habitus in the Bourdieuian sense, i.e., a set of socially learned and constructed ways to perspectivize language that challenge the previous status quo in many subfields. Central assumptions guiding the methodology and theoretical embedding are summarized in Larsen-Freeman (2006), Ellis and Wulff (2015), and Ortega (2015a). All usage-based approaches share the goal of describing and explaining linguistic patterns from observable language as it occurs in corpus or experimental data directly. Grammatical phenomena are mainly modeled in a variety of constructionist approaches, such as various strands of construction grammar (Goldberg, 1995, 2006; Croft, 2001; Sag, 2012; Boas, 2013), which are tightly intertwined with emergentist approaches to learning. Other approaches are shaped through socio-linguistic, variationist (for an overview see Geeslin and Long, 2014), and ethnographic perspectives.

Relevantly, the word *usage* can take on different scopes in different approaches and even within a single framework. Most generally, usage-based linguistics takes a behaviorist and empiricist view on language in that it seeks to describe linguistic behavior as it occurs. In modeling language acquisition, it takes the stance that language is also learned from and through usage (in emergentist/connectionist approaches). However, what constitutes usage can still differ even within this paradigm. For example, usage can be described in terms of concrete linguistic realizations (for instance by how much inflectional morphology is used) or in terms of the interactional, dialogical content of what two or more speakers experience in usage. In our research, we focus on the concrete linguistic realizations, because we have access to them more or less directly through the writing of our participants, and because we find it helpful to first document the linguistic reality as we find it in corpora, before we connect it to language-external factors.

In connectionist/emergentist models, L1-like competence is modeled as the result of a construction process using language input to arrive at linguistic abstractions and entrenchment of auditory signals as well as abstract signs (Ellis, 1996; Bybee, 2002; Hoey, 2005; Tomasello, 2009). Importantly, construction grammar traditionally poses a unified space for all types of constructions from words through morphological units to syntax, famously summarized in Goldberg's “it's constructions all the way down” (Goldberg, 2006, 18) and playfully exaggerated by Boogaart et al. (2014, 1) as “it's constructions *all the way everywhere*.” L1- and L2-learning across their linguistic (phonological, lexical, morphological, syntactic, semantic, pragmatic) levels are all equally attributed to frequency-leveraged mechanisms, and ultimate attainment in L2, including any of its limitations, is conceptualized as a function of input and usage. The native and the learner's target language systems do not differ in their underlying general quality, but in the input-dependent entrenchment of words, collocations, categories, and constructions. These are subject to constant change in both the native speaker and the learner and can be observed and analyzed in language output, i.e., experimental and corpus data^6^. Consequently, as Ortega (2013) points out, the distinction between learners and native speakers, that for a long time has so consistently been drawn even in studies dedicated to usage, becomes less and less relevant. This is also exemplified by some approaches in the area of language contact research (Backus, 2021).

The categorization and idealization of the native speaker in some of linguistic theory has been further deconstructed from a socio-historical (Bonfiglio, 2010) and sociolinguistic perspective (for instance, in a range of contributions to Doerr, 2009). Equally, the concept of nativelikeness in SLA research has been problematized from a language variability perspective by Birdsong and Gertken (2013) and others. These discussions are fueled by what has been described as a turn toward bi- or multilingualism in SLA research (Ortega, 2013; Geeslin and Long, 2014)—including the realization that multilingualism is, and has always been, the norm in language acquisition; that standardization of language is a fairly recent and often politically guided process; and that, while a speaker's language output in their various languages can be studied separately, their language system(s?) effectively cannot.

### 2.2. Previous Research Into Inter-individual Differences

Individual differences between speakers have raised attention in SLA research as factors determining the trajectory, velocity, and success of the learning process, as well as performance as a function of skill and other determining factors. Some of the observations pertain to language-internal or language-specific factors, such as shape of context, profile of the material that has already been uttered, and that is being planned (Szmrescanyi, 2006; Jaeger and Snider, 2013); language situation (Wiese, 2020); vocabulary (Kidd, 2012); or attainment as measured in production or reception/acceptability judgment (Dąbrowska, 2018; Birdsong, 2021). Much research has considered cognitive factors (e.g., aptitude, including as a function of age, cf. Berman and Nir-Sagiv, 2007), working memory, executive function, statistical learning faculty, intelligence (Skehan, 1989; Bates et al., 1995; Dörnyei, 2005; Kidd, 2012; Kidd et al., 2018) as well as more general psychological factors (attention, see, e.g., Roelofs, 2008; motivation, Lowie and Verspoor, 2019). Some consider external influences on language performance (time limit, test mode, channel, see, e.g., Ruth and Murphy, 1988; Chapman, 2016). Kidd et al. (2018) argue that individual differences result from a complex interplay of systemic cognitive and environmental factors and warn against downplaying variance in learner data as error variance, if individual differences are poorly taken into account.

In principle, all of these factors could also influence native speakers. However, where individual differences in L1 have been considered, this has mainly been done from a psycholinguistic perspective, e.g., Mulder and Hulstijn (2011), Dąbrowska (2012), and Birdsong and Gertken (2013). As a requirement for the direct comparison of L1 and L2 data, it is necessary to also gain an understanding of expectable differences among the L1 group. But L1 variability has only begun to gain awareness in L1/L2 comparison studies. For example, Mulder and Hulstijn Mulder and Hulstijn (2011) call for taking into account variability between native speakers in future SLA research, but still do this from a stratified perspective (by age; level of education). Similarly, Birdsong and Gertken (2013) discuss the necessity for a differentiation of groups in L1/L2 comparisons by consideration of inter-individual differences within and across groups. They argue (and we agree) that comparing the two groups can still be considered a legitimate method in SLA research as long as it is based on a differentiated analysis.

### 2.3. The Contrastive Paradigm

In spite of the theoretical possibilities provided by usage-oriented frameworks, variability in learner data has usually been investigated with contrastive/comparative methods e.g., *Contrastive Interlanguage Analysis* (CIA) in which a presumably homogeneous control group of native speakers is used as reference (Granger, 2002, 2015; Ädel, 2015). Effectively, even in these approaches that are sensitive to inter-individual variation, intra- and inter-individual differences in L2 data are used as indicators of the level of target language competence (Ädel, 2015; Gablasova et al., 2017). Frequencies are modeled as dependent variables or expressions of underlying characteristics, such as target language competence. This implies that frequencies in target language are distributed within predictable and stable ranges, i.e., stationary. If frequencies were not stationary in the target language, but showed high variation, an approximation to target language frequency ranges would not be possible to achieve because the target of the approximation itself would be moving^7^.

If frequency is expected to be stable and approximation to L1-like distributions is modeled as indicative of target language competence, this raises questions with respect to the adequate object of comparison. Obviously, learners cannot be expected to produce frequency distributions as they are common in newspaper or general-purpose corpora, but that is not necessarily due to lack of target language competence. Rather, newspaper or general-purpose corpora do not represent the speech of a single speaker, but are thematically and stylistically variable collections of text that are not representative of any one speaker of a language (Biber, 1993). A better object of comparison would thus be provided by the learners' input, for example through text books, assumed speech environment, and instructor speech. For example, Linford et al. (2016) investigate subject realization based on how much the assumed input and the output of their learners of Spanish match or diverge. To that end, they take a local corpus of native speakers formed under the same circumstances as their non-native-speaker corpus, and compare subject realization depending on the verb it occurs with, its frequency, and switch reference. They then examine the same measures on what they call a global corpus, Davies (2002)'s oral part of the *Corpus del español*. Indeed they find that the choice of corpus for comparison yields divergent results, i.e., that a certain distribution in the assumed input would lead to the conclusion that non-native speakers reproduce their input, while another distribution in another sample stipulated as input would lead to the conclusion that they do not, or to a different extent^8^. Although this research crucially depends on the recognition of situational variation and advocates the use of specialized corpora, it still does not consider the possibility of interference from inter- or intra-individual variation among native speakers.

In conclusion, despite the fact that many of the variables in the literature around individual differences are by no means specific to learners (Granger et al., 2015), and even though recent work has shifted the conceptualization of native speakers away from being a monolithic group, even studies that consider variation do not do so on an inter-individual level in L1 groups used for contrastive comparison.

Obviously, any speaker group characterization unavoidably carries some loss of information, since reductionist categorization implies the abstraction away from an object of study (Hulstijn, 2015). This is also the case with the group of native speakers, where, in addition to the information loss through categorization, a form of idealization tends to facilitate the assumption of homogeneity (Doerr, 2009; Davies, 2011). This may not be overall justified, as for example Dąbrowska (2012) shows considerable individual differences between native speakers of English in terms of inflectional morphology, passives, quantifiers and complex subordinating clauses. This poses challenges to the widespread idea of a definable subset of shared grammar between native speakers, which is a fundamental assumption in different theoretical strands of SLA research. Dąbrowska (2012) and DeKeyser (2012), as well as Birdsong and Gertken (2013) criticize the negligence of this fact, especially given that these differences cannot (only) be attributed to sociolinguistic factors. Birdsong and Gertken (ibid.), aside from questioning the overall comparability of monolingual native speakers with bi- or multilingual non-natives, call for careful methodological consideration of this. In the same manner, Hulstijn (2019) notes that the claim of great differences between adult native speakers serving as control groups in SLA research is still lacking a robust empirical underpinning. Our aim is to address this need for research and to illustrate native speaker variability from a corpus linguistic perspective from a group that would be predicted to behave homogeneously, following the literature.

### 2.4. L1 Variability in Learner Corpus Research

For corpus linguistics, Gries and Deshors (2014) diagnose a research deficit with respect to differences between native speakers which are used as a reference for learner language. Their analysis of the use of the modal verbs *may* and *can* in English L2 and L1 demonstrates variability among both groups. This is done with multifactorial regressions involving interactions between fifteen different factors like syntactic characteristics of the clause and various morphological and semantic features of the subject. They use a method entitled Multifactorial Prediction and Deviation Analysis with Regressions (MuPDAR), which shows statistical interactions of lexical and syntactic elements in large corpus data^9^. While the authors themselves describe this method of analysis as very complex and challenging, our work will illustrate that inter-speaker variability in L1 data can also be examined and demonstrated with less demanding analytical methods, and with smaller, more controlled and deeply annotated corpus data. This offers a more widely accessible approach to comparative SLA corpus studies, and, since smaller data can be manually annotated, allows for the analysis of a greater variety of linguistic phenomena (cf. Lüdeling et al., 2021).

According to Granger (2002), native speaker corpora provide relevant information on the frequency and use of words, phrases and structures. Occurrences and co-occurrences of certain linguistic features can be used as a basis for comparison between L1 and L2, concretely of L2 mis-, over-, or underuse (Granger, 2002; Ädel, 2015; Gablasova et al., 2017). Frequencies in L1 serve as a benchmark for the frequencies of the same features in learner language and thus play a central role in comparative methods such as the CIA in SLA research. This is a consequence of the idea of entrenchment as a direct neuronal correlate of frequency in the input. Divjak and Caldwell-Harris (2015) in a literature review present the discovery of characteristics that correlate with frequency (e.g., word length, concreteness, age-of-acquisition of a word/structure) as well as the evolution of contextualized frequency measures, such as dispersion (homogeneity of the distribution of a word in a corpus) or surprisal (how unexpected a word or sequence is, given its context). Since Langacker's (1987) introduction of the concept of usage-based learning, there has been continuing research for “the measure which is best suited to predict entrenchment” (Divjak and Caldwell-Harris, 2015, p.67). This concerns, among other things, the granularity level at which frequencies are measured along with the question of the units that are effectively entrenched (for example words, morphosyntactic categories, phonetic sequences etc., cf. for example Ellis, 1996; Croft, 2001; Bybee, 2002; Wray, 2002; Goldberg et al., 2004; Bybee and Torres Cacoullos, 2009; Ellis and Frey, 2009).

One of the few studies to our knowledge that deal with the challenges of native speaker variability in the frequency of occurrence of linguistic structures is Gablasova et al. (2017) investigation of four linguistic features in five L1 corpora of informal spoken English: a concrete co-occurrence (*I think*) and word form co-occurrences (adverb+adjective), as well as past tense and passive occurrences. They emphasize the necessity of investigating inter-speaker variation within corpora before comparing frequencies across them, because they consider it equally important to reflect on possible causes of variation between corpora, which could, for example, be due to different corpus designs, subject groups and data collection methods. The results illustrate that corpora of similar native speaker language can differ remarkably, both within and across corpora.

### 2.5. Priming and Corpus Data

So far, we have introduced relatively stable or situational factors that may lead to inter-individual differences. Those are either non-linguistic (age, region, gender); language-related (aptitude, reading experience); or fully linguistic (lexical and syntactic environment). Those affect the linguistic behavior of a speaker in generalized ways across their production (although some of them may still fluctuate over time). Another factor that affects language production is priming, i.e., the semi-persistent activation of elements that facilitates their repetition or the co-activation of other elements based on similarity of structure or content. For the purpose of this paper, priming can be understood as a mechanism that temporarily raises the probability of a word or category to re-occur after it has been introduced.

Priming or persistence started getting attention from a corpus-linguistic angle only during the past 15 years. It is at the intersection of cognition and factors inherent to the linguistic system. Its psycholinguistic underpinnings and exact mechanics are not fully understood, but the linguistic dimensions of its occurrence, as well as conditions that favor it, have been given some attention in the literature (for an overview, see Gries and Kootstra, 2017).

Priming can occur as a particular form or as a pattern (Szmrecsanyi, 2005; Szmrescanyi, 2006; Gries and Kootstra, 2017), or, as we understand it for our purposes, as lexical or structural priming, for example a morphological class rather than a specific word. If priming had an effect on the morphological level in our data, a morphological class once introduced would re-occur at higher rates than if it had not been evoked, in effect forming clusters in a text. Speakers are susceptible to other-priming (priming by external factors, such as the prompt or interlocutor speech) as well as self-priming by their own text-production. Since priming is a procedural phenomenon, its effects decrease with a higher prime-target distance. This means that it may affect *only part* of a text, making it very different from more stable factors, like age or reading experience, or even the more fluctuating, like motivation, which will still affect the *whole* text that a participant contributes. This is relevant to the methodological and theoretical model because it highlights the fact that cumulative corpus counts are not a single, but a twofold dimensionality reduction that collapses both the inter- and intra-individual variability that exists in a corpus, i.e., two ranges, into a single number.

Gries and Kootstra (2017) suggest that corpus linguistic studies are suitable for exploring priming effects, as they provide a more natural usage-based perspective on priming than psycholinguistic experiments with potentially unnatural stimuli. This specifically affects prompt-based and self-priming. Chapman (2016, p. 110) in a study of second language writing assessment shows that lexical sophistication, academic vocabulary use, syntactic complexity, cohesion, and fluency of a response can be strongly influenced by prompt characteristics. Even relatively abstract elements such as the morphological class of particle verbs in German can be prompt-primed in both L1 and L2 according to Lüdeling et al. (2017). The way writers respond to a specific prompt is also expected to have more far-reaching consequences, namely on the selected register of the produced text^10^. Although priming exists on all linguistic levels (phonetic/phonological, semantic, pragmatic, syntactic, discoursive, etc.), we will only consider structural morphological priming, which we will discuss in section 4.4.

### 2.6. Research Question

The research question guiding our analysis can be summarized as “how variable are German native speakers from a highly homogeneous group in their distribution of a) morphological subclasses of nouns and verbs; and b) higher-order syntactic elements in task-specific, highly controlled corpus data?”, or, simpler put “what kind of information with respect to inter- and intra-individual variation would we lose in the cumulative analysis of our corpora?”

We enter from a learner corpus-oriented research paradigm, but we will not look into learner data in this study—instead, the observations we report are born from *intended* comparisons with learners within a connectivist and emergentist usage-based framework.

## 3. Materials and Methods

The texts used in our study were written by participants of the native speaker control group in the collection of the two German learner corpora Kobalt (Zinsmeister et al., 2012) and Falko (Reznicek et al., 2012). Both corpora are comprised of prompted argumentative essays written under controlled conditions (90 min, handwritten or typed without aids such as dictionaries). Kobalt contains 20 L1 texts, in Falko we use 95 L1 texts for the morphological analysis and 65 for the syntactic categories. We are forced to accept this limitation since not all L1 texts in Falko are available with corrected dependency tags yet. Neither of the corpora was compiled for the purpose of this study, both are publicly available (see data availability statement at the end of this paper) and have been previously used in a number of other studies (Hirschmann et al., 2013; Zeldes, 2013; Hirschmann, 2015; Lüdeling et al., 2017, 2021; Shadrova, 2020; Wan, 2021, among others).

L1 contributors to both corpora were chosen from a very homogeneous group, 12th year high school students from the same school in Berlin in the Kobalt subcorpus, and early college students from Berlin as well as high school students from Berlin and Potsdam (a smaller city near Berlin) in the Falko subcorpus. This way, we were able to control for age, region, urban vs. rural influences, and even exposure to the same teaching materials in the case of high school students. We did not control for socio-economic status directly, although both high schools were chosen from more affluent parts of town for practical reasons. Unfortunately, the reality of the German education system is highly selective and stratified. We do not expect that there would not be any differences at all between our participants or their parents with respect their socio-economic status or education background. However, based on German population statistics, we can assume a high level of homogeneity based on the group selection and the social reality in Germany^11^.

Both corpora are prompt-based and controlled with respect to topic. In Kobalt, the prompt is *Geht es der Jugend heute besser als früheren Generationen?* “Do young people today do better/have a better life than previous generations?” In Falko, participants were free to choose from four different prompts on topics attempting to elicit a discussion of controversial points of view. The topics that were chosen for corpus collection resemble the ones used in the ICLE corpus, cf. Granger et al. (2020).

- *Kriminalität zahlt sich nicht aus*. (“Crime does not pay off”, labeled *crime*);
- *Die meisten Universitätsabschlüsse bereiten die Studenten nicht auf die wirkliche Welt vor. Sie sind deswegen von geringem Wert*. (“Most university degrees do not prepare students for the real world. They thus are of low value,” labeled *university*);
- *Die finanzielle Entlohnung eines Menschen sollte dem Beitrag entsprechen, den er/sie für die Gesellschaft geleistet hat*. (“A person's financial remuneration should depend on the contribution that they make to society,” labeled *incentive wage*);
- *Der Feminismus hat den Frauen mehr geschadet als genutzt*. (“Feminism has done more harm than good to women”, labeled *feminism*).

Neither elicitation was based on school work or homework or graded in any way. Participants contributed texts of variable length. In Falko, text lengths range from 181 to 1728 tokens including fluctuations by topic (min. 217, 284, 181, 436; max. 1728, 1305, 1335, 1184 tokens for the topics crime, feminism, incentive wage, and university respectively; mean: 822.20, 886.46, 872.17, 871.88; median: 712, 915, 846, 978). In Kobalt, text lengths range between 483 and 813 tokens (mean: 624.45, median: 644.5).

Both corpora contain metadata on the participants' linguistic background (language biography, i.e., L1s and L2s with age at the onset of acquisition, years of training, years of immersive exposure). These were identically collected in the L2 subcorpora of both corpus projects, but are highly uniform in our L1 subcorpora, with barely any early bilingual speakers and no longer interruptions of L1 immersion. Kobalt additionally contains scores from a standardized c-test (onDaF, now onSET, Eckes, 2010). We did not find correlations between the frequency of morphological forms including a binary distinction between complex vs. simplex forms on the one hand and gender or high school vs. college students (i.e., level of education, self-selected group of language students) on the other. No other correlations were found with other aspects of the available metadata either. We will hence not address this issue further.

### 3.1. Methods

We present descriptive statistics, using relative frequencies (normalized to all occurrences of verbs in Kobalt and nouns in Falko) and proportions of categories normalized to 100%.

We computed regressions for potential text length dependency, since text length is well-known to correlate with many corpus linguistic measures. In our data, text length correlates highly with simplex verbs and nouns, but not with any of the other categories (see section 6.1 in the Appendix). We include plots of randomized samples of the original lengths in the appendix to show the expected variance if categories were randomly distributed, confirming our conclusion that text length is, somewhat surprisingly, not a meaningful factor in morphological category distribution.

With the exception of regressions for text length, we limit our statistics to basic descriptive measures such as percentages and simple variance computations, since we are mainly interested in the composition of categories from subclasses. Accounting for the variance of several factors in a system in a single measure necessarily involves a dimensionality reduction that we are not ready to perform on this data, because we have limited understanding of its linguistic repercussions. In addition, from the results we obtain in the comparison between native speakers, we cannot be sure that frequencies converge. This limits our trust in the abstractability of relative frequencies from this data to idealized probabilities—we are not confident in that the data is ergodic and stationary (Shadrova, ress; Piantadosi, 2014; Dȩbowski, 2018), or can truly be seen as a random sample from a population in the statistical sense. If it were not, the central limit theorem would be caused to fail and inferential statistics would be rendered undefined. More clarification of the mathematical underpinnings of those categories as they occur in corpora are required before we can proceed with inferential statistical modeling, such as regression. This remains for future research.

We further present a sliding window analysis for a discussion of priming as a factor that could potentially contribute to high variability. For this, we have defined overlapping windows of 50 tokens each, the first covering tokens 1-50, the second 2-51, the third 3-52, and so on. Each text is represented by *textlength*−49 windows. Data points show cumulative counts of the occurrence of the respective category in each window. Colors differentiate between the total token occurrence of the category and the number of different lexemes (types). For example, a category can be represented in 5 tokens and 3 types within 50 tokens, i.e., one type would be repeated three times, or two would be repeated twice in that window. For most of the windows, the number of types equals the number of tokens. Window size was chosen arbitrarily, but attempting to maximize representation of peaks and slumps. If window size is chosen too large, two peaks might be bridged, making it appear as though the category was uniformly represented across the whole window. If window size is chosen too small, accumulations are not properly represented. A better understanding of correct choice of window size should be derived from future research in alignment with psycholinguistic observations.

All analyses were performed using R (R Core Team, 2015) on RStudio (RStudio Team, 2015) with packages dplyr (Wickham et al., 2018), reshape2 (Wickham, 2007), and ggplot2 (Wickham, 2016).

### 3.2. Annotations and Categorization

We investigate structural variation on several levels of complexity and abstraction as we expect that the amount of linguistic material involved in a structure may influence the range of variability. As representatives of a higher level of interdependent structure, we examine syntactic dependencies and part-of-speech distributions. For more fine-grained categories, we look at the morphological and morphosemantic subclasses of nouns and verbs.

Both corpora are part-of-speech-tagged with TreeTagger (Schmid, 1994) and the Stuttgart-Tübingen tagset (Schiller et al., 1995), and dependency-parsed with manual correction of dependencies (MaltParser, Nivre et al., 2006 with Foth, 2006's dependency grammar). The part-of-speech tagging and dependency parsing are generated on the target hypothesis, a normalization layer that consists of a hypothetical reconstruction of an orthographically and syntactically correct version of the text (Reznicek et al., 2013). Lexical items are not corrected or changed except for orthography. This method was designed for L2 data, but even for essays written by L1 speakers, automatic parsing does not yield satisfying results when based on the original document, hence the need for a normalization layer.

Morphological categorizations of nouns (Falko) and verb-type classifications in terms of syntactic category and morphosemantic components (Kobalt) were manually annotated. Detailed annotation schemes for both classifications can be found in the Appendix in Supplementary Materials^12^.

Nouns in Falko were classified according to the word formation processes underlying their structure, for example as determinative compounds, derivations, nominalizations, etc. The annotation followed the guidelines in Lukassek et al. (2021) that were developed in several iterations of test annotations by two or more annotators, discussions of the results and refinements. Guidelines were furthermore tested by three independent annotators whose inter-annotator-agreement (Artstein and Poesio, 2008) for the annotation layer reported in this paper was perfect (Fleiss' κ = 0.81).

Lexical verbs in Kobalt were classified with respect to their morphosemantic properties (simplex vs. complex, i.e., particle or prefix, vs. support verbs). More detailed information on these classes will be provided in the next section. Syntactic verbs were classified according to the syntactic environment they trigger (modal, modifying, auxiliary, copula, constructional verbs). Simplex, particle, prefix, modal, modifying, auxiliary, and copula verbs are easy to classify because they occur in very clearly defined syntactic environments or have a distinct shape (prefix, particle, simplex verbs). Support verbs and constructional verbs are subject to more ambiguity, since they mark a deviation from the semantic or syntactic norm. More detailed information on these annotations can be found in Shadrova (2020, section 3.2) and in a Zenodo repository which also contains the annotated data: 10.5281/zenodo.3584091.

## 4. Results

### 4.1. Kobalt: Verb Subclasses

We first investigate the distribution of subclasses of verbs in Kobalt. For this, we will look into morphologically and syntactically defined subclasses. For the syntactic subclasses, we consider auxiliaries, copula verbs, modal, and modifying verbs, as well as verbs in constructional use (see Appendix in Supplementary Materials for annotation guidelines)^13^. Morphologically complex verbs in German include prefix verbs that contain an inseparable prefix to a base such as *verlegen* (“misplace” vs. the simplex *legen* “to put, to place”) and particle verbs, that include a separable particle to a base. The particle is split from the base in inflected verb forms, i.e., in non-analytical constructions (constructions lacking an auxiliary or modal verb), and forms a different participle. In the case of the particle verb *vorlesen* “to read out loud, to read to someone” vs. the prefix verb *verlegen* “to misplace,” this occurs in following way: *Sie liest den Kindern die Geschichte* ***vor****; Sie hat den Kindern die Geschichte vor****ge****lesen* “she is reading/has read the story to the children” vs. *Er* ***verlegt*** *oft seine Brille; Er hat seine Brille* ***verlegt*** *(not:* ***vergelegt****)* “he has misplaced/frequently misplaces his glasses”^14^. Semantically complex verbs here refer to the difference between simplex verbs on the one hand and support verbs in support verb constructions (*Funktionsverbgefüge*), which take on a non-compositional, non-literal meaning in lexicalized VP-NP combinations, on the other. Morphologically complex verbs can also be considered semantically more complex because they tend to semantically extend their bases^15^.

In our analysis, we are interested in the *composition* of the verb class with respect to its syntactic subclasses, not simply in the relative frequency of each subclass—how much space does each subclass take relative to the other categories^16^?

**Figure 1:**
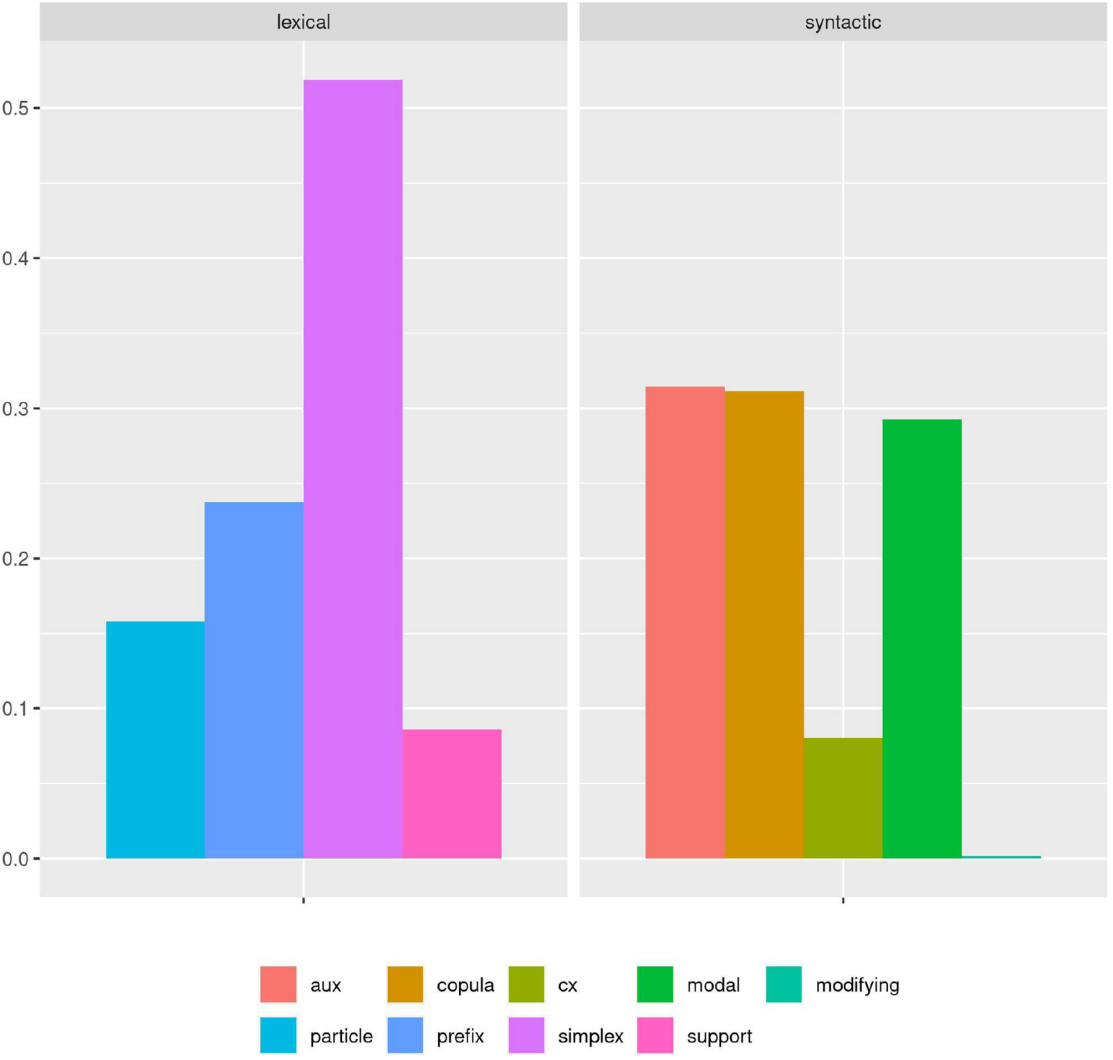
A simple bar plot of cumulative proportions of each verb subclass shows a clear ranking among lexical verbs, with simplex verbs covering more than twice as many cases as particle and prefix verbs. Among syntactic verbs, modal, copula, and auxiliary verbs are nearly equally represented in the cumulative view.

As we would categorize the phenomenon according to our guidelines, we would find a distribution as visualized in Figure 1. From this result, we could derive conclusions for our hypothesis—for example, that auxiliaries, copula verbs, and modal verbs are equally frequent; and that simplex verbs are the most frequent category, followed by prefix and particle verbs—and we could bring that together with SLA theory to hypothesize how those distributions might diverge in learners.

**Figure 2:**
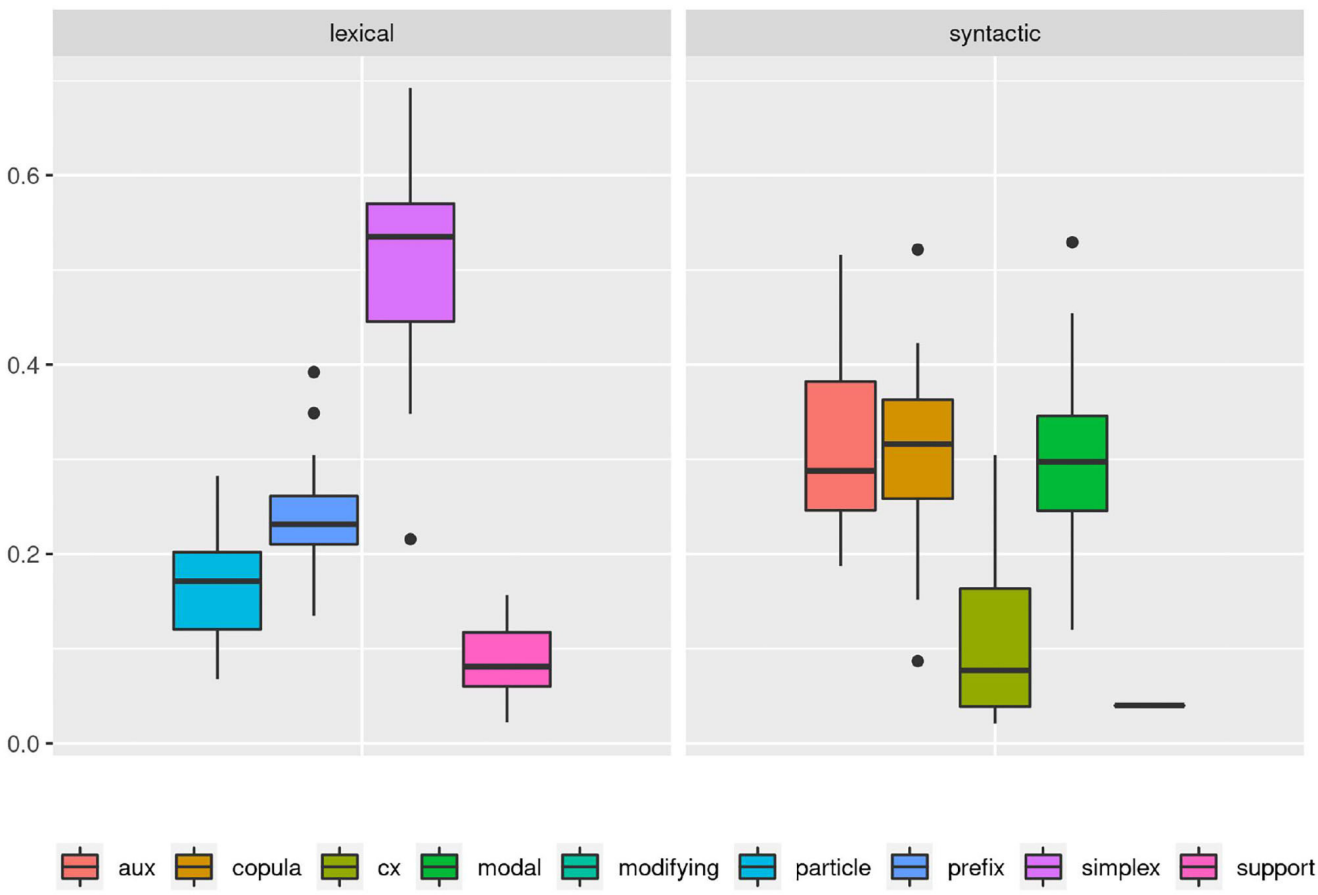
A boxplot based on the proportion of each verb subclass as it is found in the individual documents of the corpus shows a more complex picture. Long whiskers indicate considerable overlap between all syntactic verb subclasses. Similarly, the non-simplex lexical verb subclasses all partially overlap and even the clear dominance of simplex verbs among the lexical verbs is called into question with several outliers overlapping between prefix and simplex verbs.

Or could we? The boxplot in Figure 2 accounts for the variance between documents in each category. Here we can see that there is in fact considerable variation within and overlap between categories. While on average, the previous description still holds true to a degree, it no longer covers all of the data. However, even from this perspective we can still model category frequency to fit with the idea of an idealized, albeit strongly probabilistic native speaker.

**Figure 3:**
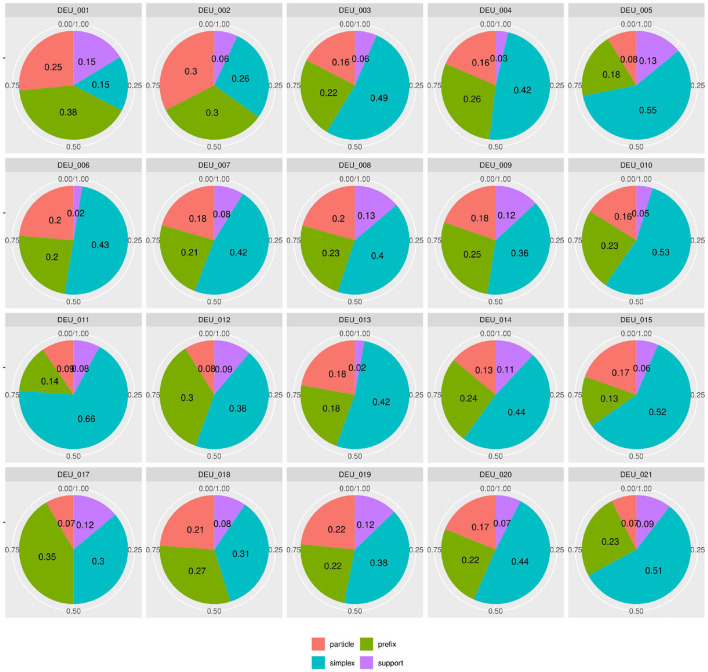
Individual distributions of lexical verb subclasses in the native speaker documents in Kobalt show striking differences in proportions. Pie charts are usually not ideal tools for distribution visualization, but were in our view the best choice in this case, cf. footnote 18.

But since usage-based theory presumes frequencies to be meaningful and reasonably stable aspects of linguistic expression, this wide frequency range raises our suspicions - what is going on in the L1 data and how do we consider it methodologically? The composition of subclasses in each text, represented in Figure 3, provides a clearer picture of the vast variability in frequency realizations in what would theoretically be a homogeneous L1 corpus^17^. Rather than just using “more” or “fewer” complex forms, each individual participant in the native speaker group appears to follow their own *distribution* of classes—a type of information that is, to a degree, implicitly included in the boxplot in Figure 2, but becomes strikingly more obvious in the tiled pie charts. While some participants use prefix verbs more than any other category (DEU_001, DEU_017), others use twice as many simplex verbs as all other types combined (DEU_005, DEU_011). Some use more support verb constructions than particle verbs (DEU_005, DEU_012, DEU_021), while for most others, support verb constructions make up the smallest part. Since quantitative corpus linguistics builds on the assumption that frequency of occurrence has meaning, this result is puzzling and slightly worrisome. Which one of those speakers should be considered representative of the target language for a learner?

**Figure 4:**
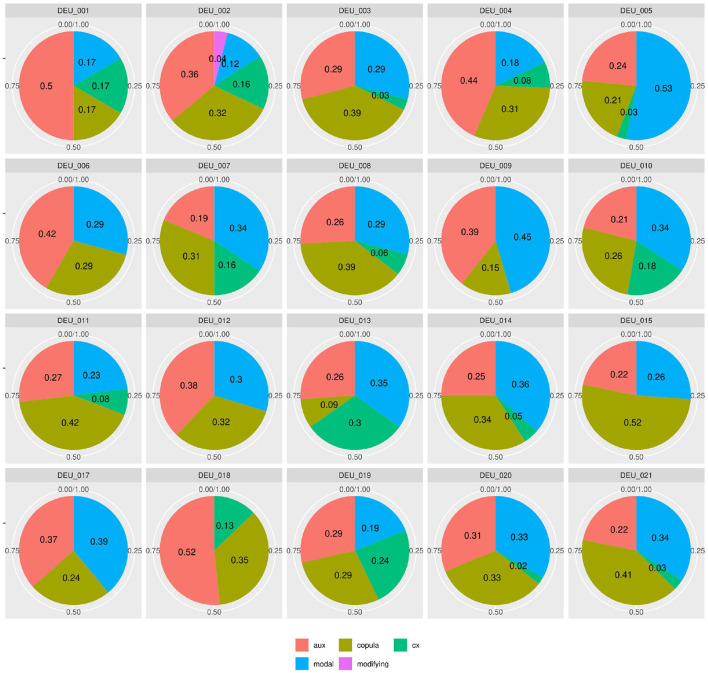
Individual distributions of syntactic verb subclasses in the native speaker documents in Kobalt show striking differences in verb subclass proportions in spite of low degrees of lexical semantics present in the categorized verbs.

Figure 4 shows similar diversity in the distributions of syntactic or functional verbs, i.e., verbs that occur in or trigger specific syntactic environments, such as auxiliaries, copula, or modal verbs. If speakers followed frequency distributions in their realization of words (by morphosyntactic category) or relational structures (to express modality or temporality), modals and auxiliaries should be distributed more equally. For instance, modal verbs are a) very schematic and transparent in their use and b) not very diverse^18^. Auxiliaries are even more limited and equally transparent. However, in our data, the proportion of auxiliaries among syntactic verb forms lies anywhere between 19% (DEU_007) and 52% (DEU_018). Even more strikingly, the use of modal verbs among the morphosyntactic subclasses ranges between 0 (DEU_018) and 53% (DEU_005). If the same was found in a learner group, one might conclude that a learner avoids modal verbs due to incomplete attainment, but obviously, in a native speaker at high school level, this explanation is lacking.

### 4.2. Falko: Noun Morphology

**Figure 5:**
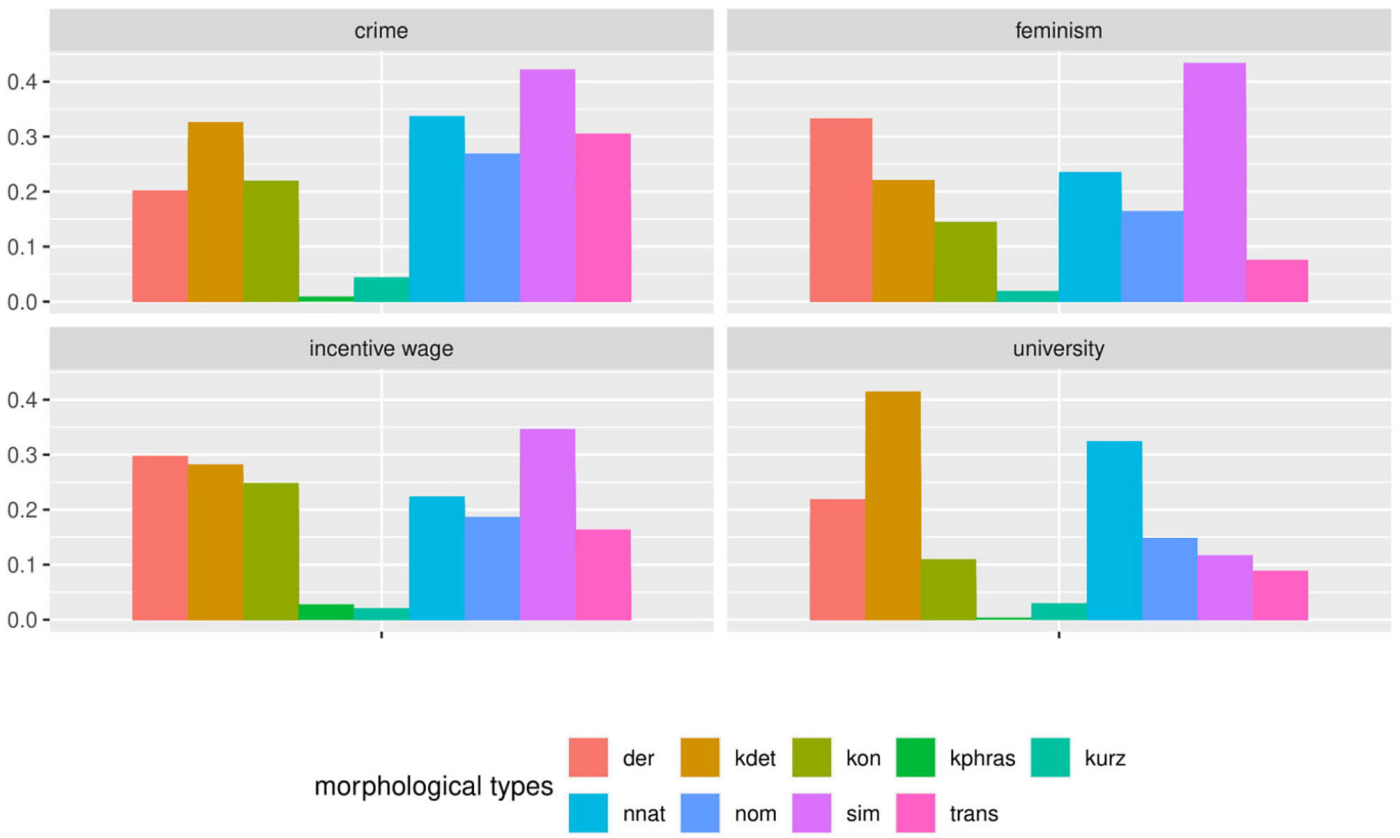
The cumulative distribution of morphological types of nouns across Falko differs by topic. For instance, in the *feminism* topic simplex nouns are prevalent, whereas in the *university* topic non-native nouns and determinative compounds are the largest groups.

**Figure 6:**
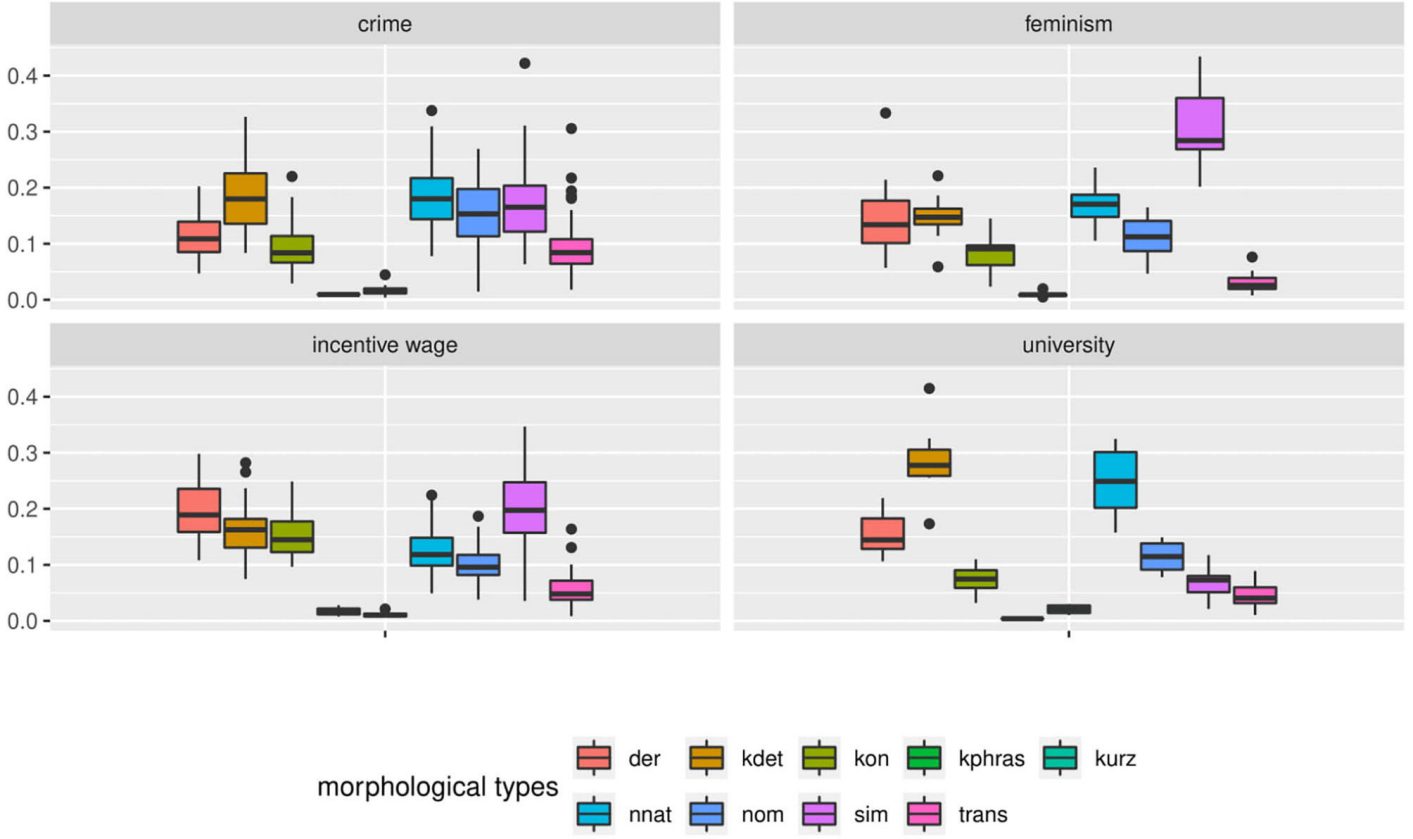
The frequency of morphological types of nouns in Falko varies greatly when the distribution across individual documents is considered. Even some of the most frequent types, like simplex nouns *sim* in the incentive wage topic, barely occur in some texts. It also appears as if certain topics elicit more clearly defined distributions (*university* and *feminism*), while the overlap between boxes is much larger in the other two topics, particularly so in the *crime* topic. This may be due to higher random variability or an artifact from a lumping of different speaker, register, or text type typologies.

In a similar fashion to Figures 1–3, 5, 6 show the distribution of noun morphology in the Falko corpus. We first see a bar plot showing the cumulative distribution of morphological types of nouns across the corpus in Figure 5 and then a box plot accounting for the variance between the 95 native speaker documents included in Falko in Figure 6. Both plots are divided by topic, because the topic may influence the chosen text type or register of the text, which in turn may trigger variability in linguistic realization. Obviously, in a text written in response to the prompt on *feminism*, we would expect a significant amount of nouns referring to adults of either female or male gender and to children. All of these concepts are realized as simplex nouns in German (*Frau* “woman”, *Mann* “man”, *Kind* “child”). Furthermore, the topic is introduced with a prompt, which in an analysis of morphological aspects of complex verbs in Falko has been shown to produce structural priming effects on the morphological level (Lüdeling et al., 2017)—both learners and native speakers use more particle verbs if the prompt includes a particle verb. At least for the university topic, the prompt yields a similar effect for nouns. The prompt features two non-native nouns, one of which is part of a compound. From Figure 5, we can see that *kdet* (determinative compounds) and *nnat* (non-native nouns) are the two most frequent classes, which we interpret in terms of a priming effect.

The document-wise distribution in Figure 6 yields a more differentiated picture. Let us consider simplex nouns as an example. According to Figure 5, this noun type is prevalent in the *feminism* topic. However, in Figure 6 we can see that individual texts include fewer simplex nouns than derivative nouns, which constitute only the fourth most frequent noun type in the cumulative distribution of the *feminism* topic. In the *incentive wage* topic, simplex nouns are one of the two most frequent noun types in the cumulative distribution. Nevertheless, we can see from the document-wise distribution in Figure 6 that simplex nouns rarely occur in some of the texts.

**Figure 7:**
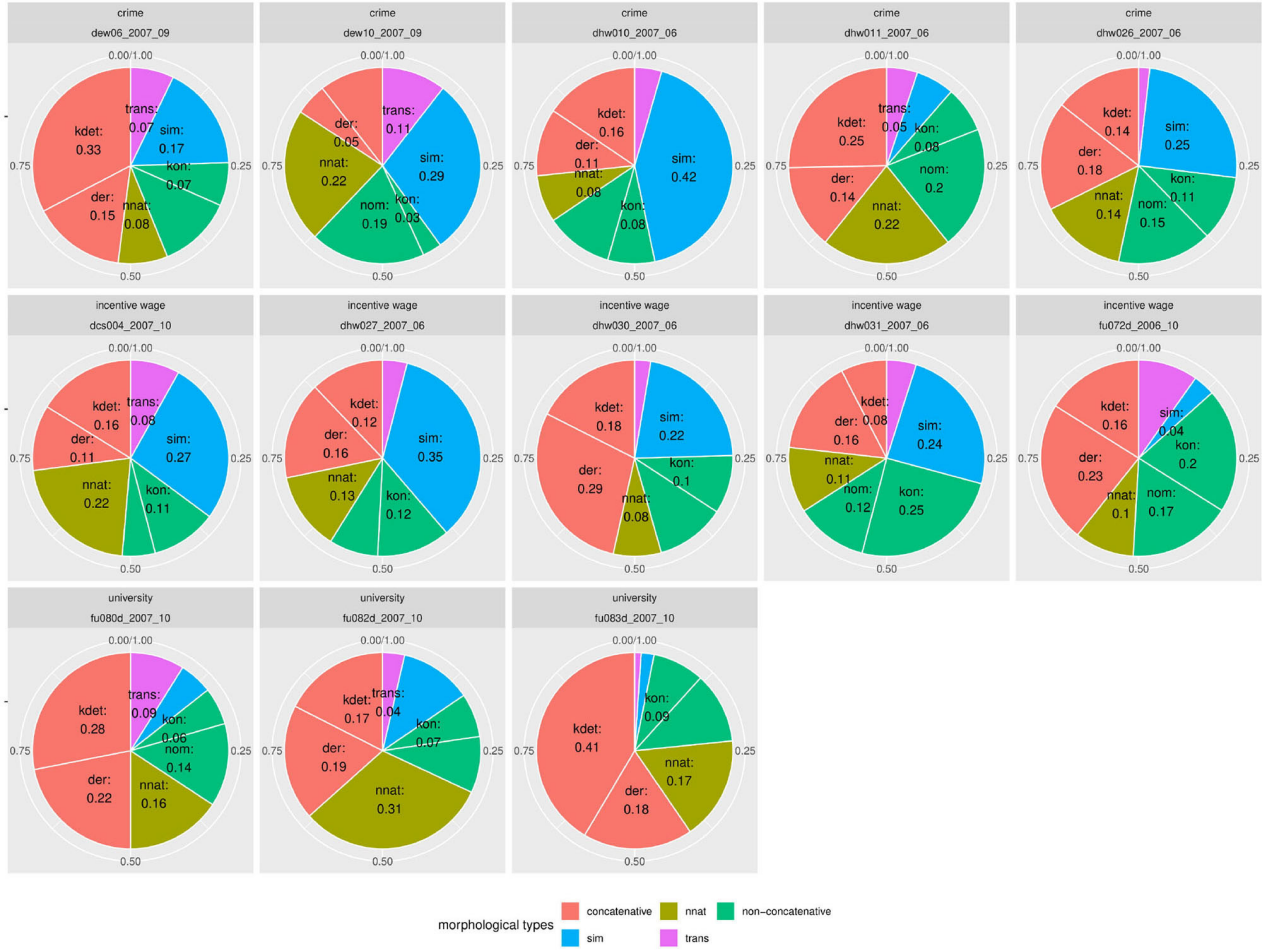
The variety of distributions of noun types is even more striking when displayed by individual document. Even documents pertaining to the same topic vary greatly.

Figure 7 shows document-wise distributions of each noun type. For better interpretability, we grouped the two concatenative word formation types compounding (*kdet*) and derivation (*der*) as well as the two non-concatenative types conversion (*kon*) and other nominalizations (*nom*). Due to space limitations, we only present selected distributions in Figure 7. Plots for the remaining texts can be found in a Zenodo repository (10.5281/zenodo.4752308). Within the texts from the *university* subcorpus, the differences for the concatenative class are most striking. Whereas in text fu082d_2007_10, concatenative word formation processes account for 36% of all nouns, in fu083d_2007_10, the concatenative group covers 59% of all noun occurrences. Similarly, non-native noun formation varies between 16 and 32% of all nouns in the respective texts (cf. fu080d_2007_10 vs. fu070d_2007_10).

A similar variance can be observed for the texts on *incentive wage*. As we can see from the document-wise distributions, the simplex nouns (*sim*) vary between 4% and 35% (cf. fitfu072d_2006_10 and dhw027_2007_06). Concatenative nouns (*der* and *kdet*) make up between 24% and 47% of all nouns (cf. dhw031_2007_06 and dhw030_2007_06). Non-concatenative nouns (*kon* and *nom*) vary between 16% and 37% (cf. dcs004_2007_10 and dhw031_2007_09). Between these extremes, varying sub-divisions of the noun spectrum are possible.

The strongest variance in distributions can be found in the subcorpus of texts on the *crime* prompt. Transpositions (*trans*) account for 2% to 31% of all nouns (cf. dhw026_2007_06 and dhw022_2006_06). Concatenative nouns (kdet and der) vary between 16% and 48% (cf. dew10_2007_09 and dew06_2007_09). Simplex nouns are being used between 6% and 42% of all occurrences (cf. dhw011_2007_06 and dhw010_2007_06).

In a nutshell, the distribution of morphological nouns in Falko shows that deriving insights about the frequency of noun classes from data accumulated over speakers is highly problematic. The fact that one class is prevalent in the overall distribution does not mean there cannot be individual texts with entirely different relative frequencies for the same class. This raises the question whether cumulated speaker data, at least for this phenomenon, is interpretable at all, or in other words, whether even situationally specified target language frequencies can be defined in the first place.

### 4.3. Syntactic Classifications Affecting the Larger System

**Figure 8:**
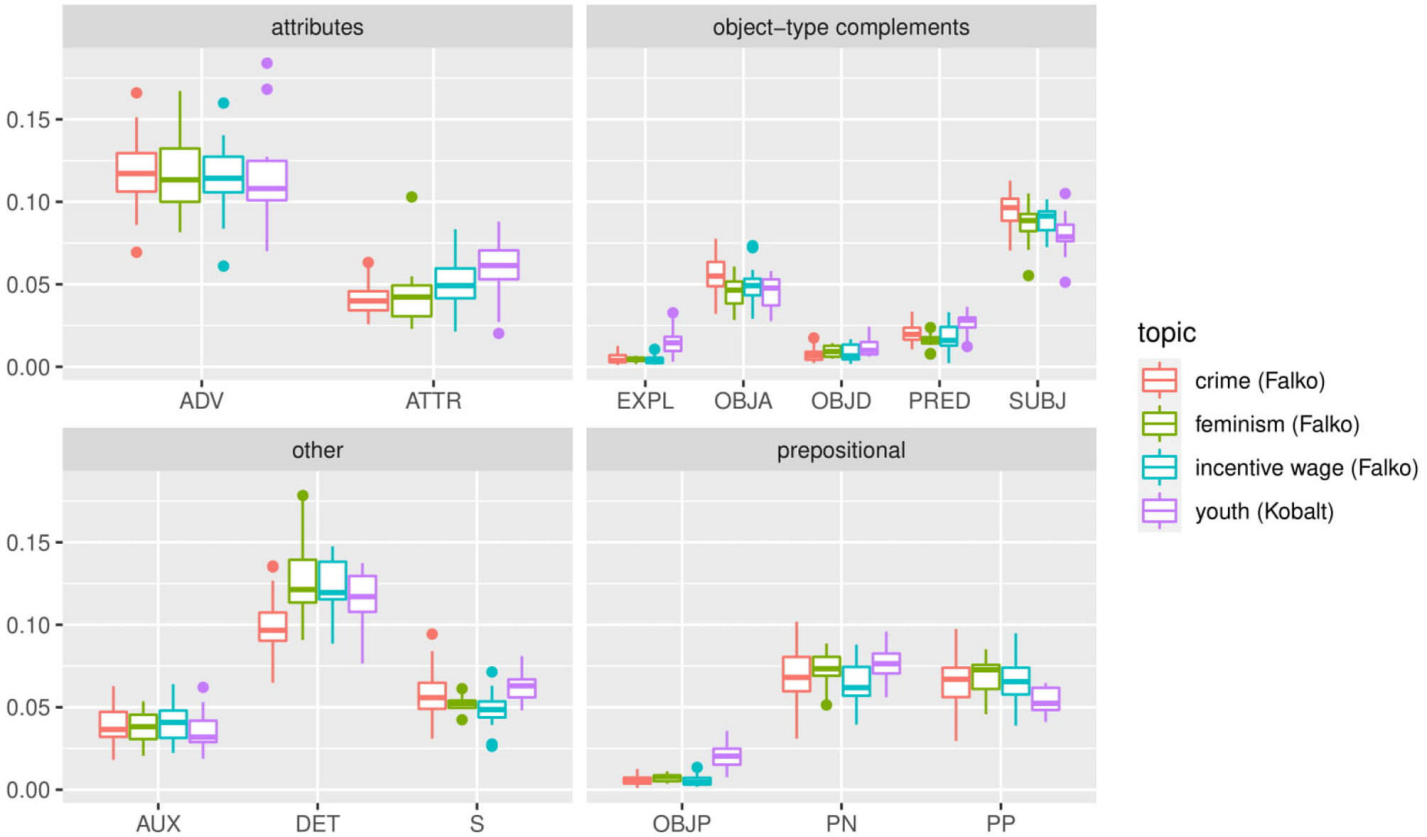
Distribution of frequent dependency types in Falko and Kobalt relative to text length. Dependencies included: adverb and attribute (attributes), prepositions in free PPs and prepositional objects, nominal compliments to PPs (prepositional), subjects, predicates, accusative and dative objects (object-type complements), determiners, lexical, and auxiliary verbs (other). Although frequencies are overall more stable compared to morphological subclasses of verbs and nouns, there still are noticeable differences by topic/corpus (more attributes, prepositional objects, lexical verbs (S) in Kobalt; fewer determiners, more subjects in the *crime* topic in Falko. This might be due to differences in lexical choice or in register realization.

**Figure 9:**
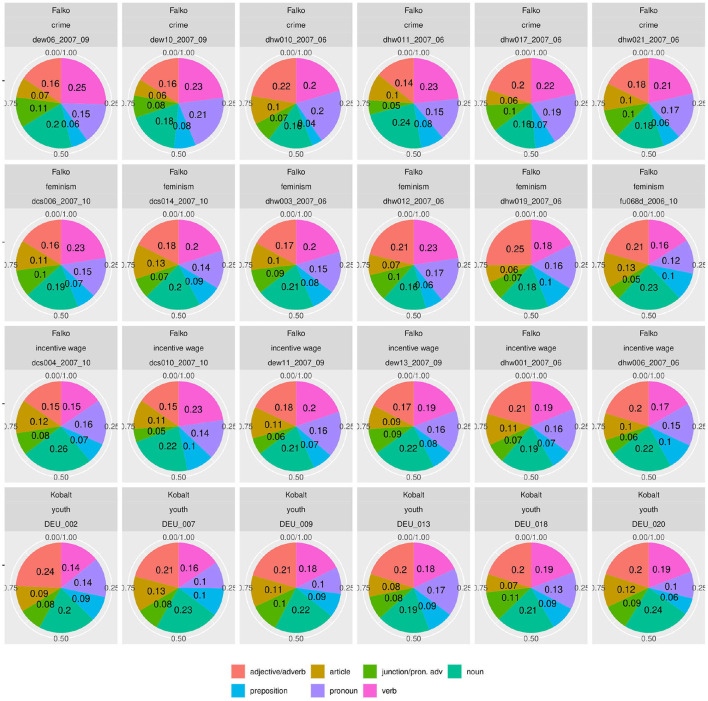
Individual part-of-speech distributions in Falko and Kobalt are much more homogeneous than the morphological, syntactic, and morphosemantic subclasses of verbs and nouns.

However, such differences do not appear across syntactic categories. Figures 8, 9 show the distribution of parts of speech and syntactic dependencies in randomly selected texts from Falko and Kobalt^19^. Unlike the previous analyses, these plots show much more comparable realizations of category proportions. That is not to say that there is no variation at all—in fact, there is at least one text in the individual dependency distribution in Falko that sticks out with a much lower proportion of prepositional dependencies (dhw_010_2007_06, top row third from left) than any other text shown here. There is also some fluctuation in the proportions between the other types. However, overall, for most texts, distributions are roughly quartered between the four categories, or rather tend to be realized through attributes and object-type dependencies by about half, filling up the other half with 40/60 prepositional and other (verb and determiner) type dependencies. Figure 8 shows that there are also some topic effects.

**Figure 10:**
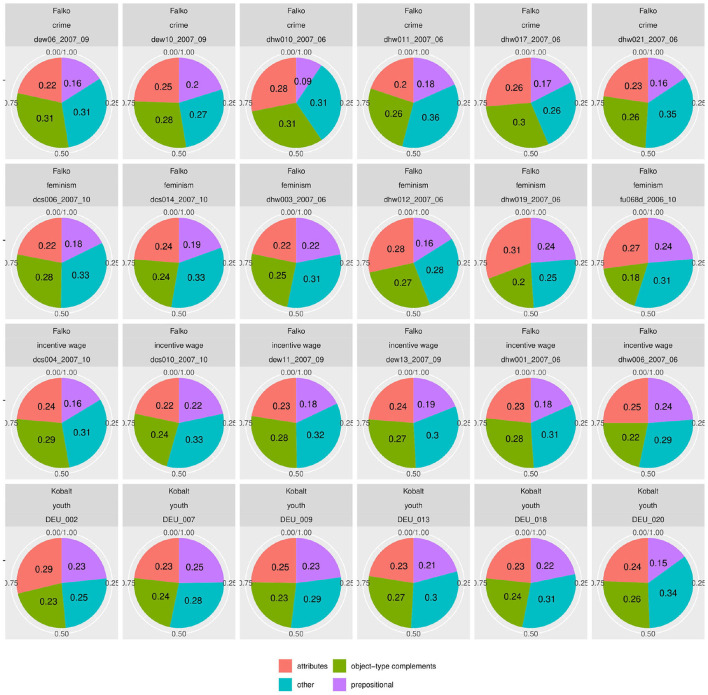
Individual distributions of frequent dependencies in Falko and Kobalt documents. Dependencies included: adverb and attribute (attributes), prepositions in free PPs and prepositional objects, nominal complements to PPs (prepositional), subjects, predicates, accusative and dative objects (object-type complements), determiners, lexical, and auxiliary verbs (other). Proportions of dependency types to one another are much more homogeneous than morphological subclasses of elements. One exception is dhw_010_2007_06 (3rd from the left in top row), which contains only 9% of prepositional dependencies (prepositions and nominal complements to PPs).

Similarly, parts of speech are distributed more equally between texts (Figure 9). This is not surprising, trivially following from Figure 10 because dependencies are derived from parts of speech, but also because part-of-speech distributions are known to be language-specific with such clarity that they can be used for determining the native language of competent L2 speakers writing in their second language, and even the original language of a professionally translated text (Teich, 2003). In fact, the success of statistically based language parsing and translation is based on the observation that (some) linguistic categories follow specifiable distributions. Against this background, it is interesting that we still find differences in these plots, both by individual distributions and by topic and corpus. However, these are nowhere nearly as pronounced as those in the subclasses of verbs and nouns. What could explain the even larger variability in the realization of morphological, morphosemantic, and syntactic categories in our corpora then?

### 4.4. Priming and Self-Priming

**Figure 11:**
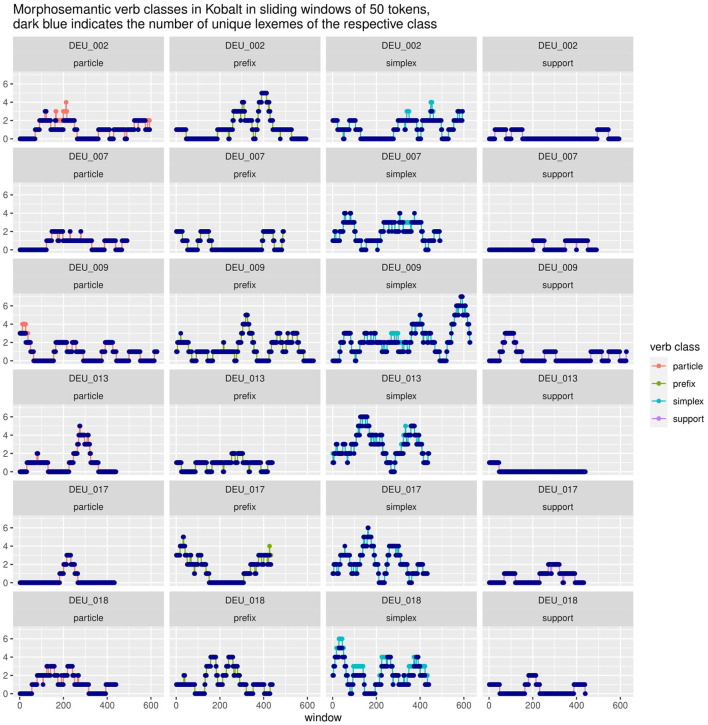
Strong clustering effects can be seen for prefix (DEU_002, DEU_009, DEU_017) and particle verbs (DEU_013, DEU_017). However, not all speakers exhibit those to the same degree (DEU_007). Lexeme repetition or α-priming does not appear to play a significant role.

**Figure 12:**
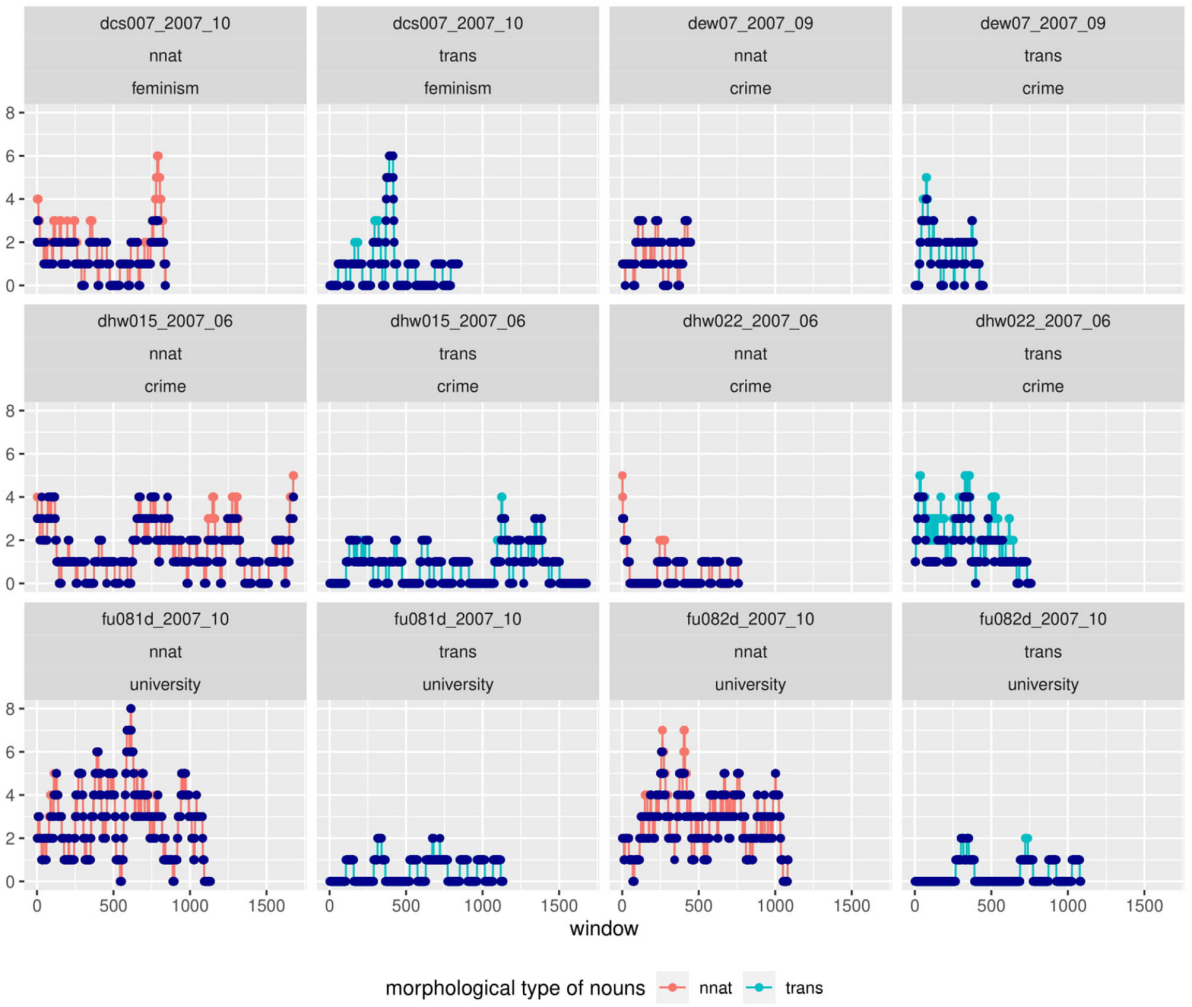
A sliding-window analysis of nouns in Falko shows the clustering of categories in some, but not all texts. Morphological categories of nouns can strongly interact with topic, as in non-native nouns in the *university* topic. The repetition of lexemes within windows (suggestive of α-priming) is less likely than the introduction of new lexemes of the same type.

Results so far have shown that cumulative corpus counts do not do justice to the internal distribution of the corpus, but that within a corpus, inter-individual variability needs to be accounted for. We further suspect that even a cumulative count of categories across an individual text marks a dimensionality reduction that could hide some of the underlying dynamicity. We will therefore look into the role of priming in our phenomena. For this, we are going to take a closer look at distributions of specific morphological categories in course of the texts. In Figures 11, 12 we present data from a sliding window analysis of selected texts in Kobalt and Falko^20^. Each data point represents the number of elements of the respective category within a window of 50 tokens, for example 3 particle verbs within 50 tokens (words and punctuation). The first window spans tokens 1-50, the second 2-51, the third 3-53, and so on. There are *text length - 49* windows for each text.

If a category occurs once, the count stays at one until the windows have slid by its first occurrence. Thus, if a category occurs several times, the peak remains until the window slides past the *first* occurrence. A peak can persist over many windows if the first occurrence drops out but is replaced by another occurrence at the higher token end of the window. If a category is distributed equally or irregularly over the text, the line should be erratic: it is counted once or twice, then drops out, then occurs once or twice again. In many cases, however, we find peaks of five or even six occurrences of a category within a 50 token window^21^. This can be due to lexical repetition/recurrence, which is why we provide the number of unique lexemes for each category within the windows (marked dark blue in the plots). For most data points, their total occurrence overlaps with the number of lexemes, i.e., each occurrence represents a separate new word, not the repetition of previous words within that window^22^.

Lexical recurrences would indicate lexical priming. Overall, we do not find strong evidence for this, although there are some cases. If there are many lexically diverse occurrences, that can indicate structural priming: once participants start using a structure, they stick with it, until they prime themselves to another category. We see strong evidence for this in the case of morphosemantic verb categories in Kobalt in Figure 11. Each row represents the four morphosemantic categories (particle, prefix, simplex, and support verbs) of an individual speaker, with data points representing the number of occurrences of each category in each window, similarly to a time series plot. Several texts show high peaks of a category, for example up to five prefix verbs within a 50-token window in texts DEU_002, DEU_017, and DEU_009. Perhaps even more intriguingly, it appears that there is a progression between forms, i.e., that speakers peak in one category and then move on to the next. This happens for instance in DEU_002, which features a range of windows with 3-4 particle verbs, followed by two peaks in prefix verbs; or in DEU_017, which begins with a number of prefix verbs, then introduces three particle verbs within a small number of windows—which are also the only three particle verbs in this text—and then returns to a peak in prefix verbs. Simplex verbs show more erratic curves, which might be due to their overall higher frequency or due to category conflation (perhaps certain types of simplex verbs prime for similar types that cannot be distinguished under the general *simplex* label). However, even simplex verbs interact with the other curves, for example in DEU_013, where the text begins with a high number of simplex verbs, which then make room for a peak in particle verbs, and then returns to a second peak in simplex verbs.

Figure 11 also shows that not all speakers are equally susceptible to clustering effects in morphological structure: DEU_007 does not show striking effects in particle, prefix, or support verbs; and DEU_018 shows nearly parallel curves for particle and prefix verbs, peaking twice at 3 vs. 4 occurrences respectively within a small range of windows. The number of unique lexemes closely follows the curves in all categories except simplex verbs in nearly all cases (with the exception of some particle verbs in DEU_002 and DEU_009). This suggests that the differences in the proportions of subclasses of verbs do not stem from different degrees of lexical richness of repetitive style. However, this does not conclusively mean that all forms are primed morphologically (structurally). It is possible that there is partial lexical priming through either the verb base or the prefix or particle, i.e., paradigmatic lexico-structural priming. This lies outside of the scope of this paper and will be treated separately in future research.

While there appears to be convincing evidence for priming or clustering effects for verbs in Kobalt, the case is more complicated for nouns. First of all, nouns vary more than verbs, both in the lexicon and each text, so that it is more difficult to set a baseline for when to assume a priming effect—each noun will belong to a morphological category, and since there are many, a number of each is to be expected in each window. Secondly, noun morphology is less transparent than verb morphology, and for some categories, structural properties are very abstract. This is the case for example in compounding, where the structure consists of only the combination of two words and headedness; or in transposition, where the structure is the use as another syntactic category rather than changes to the word itself. Unlike this, complex verb morphology, at least in the case of particle and prefix verbs, has a more distinct and obvious shape that speakers are likely more aware of (prefix/particle + base; plus phonetic features) or from which it is easier to draw connections to other forms. Compounding seems less restricted, it is hard to tell whether the form [noun + noun] was primed from a single noun or a compound. This requires a more detailed and qualitative analysis, which we will provide in a separate paper at a later time.

A clustering of categories can also be due to the coordination (listing) of elements, which is typical of some topics in Falko. For example, in the *university* topic, participants frequently mention a number of university programs such as biology, chemistry, psychology, etc., which in German tend to be of neo-classical origin (labeled as non-native). It is difficult to distinguish between this case and structural priming in less obviously related contexts without taking more qualitative evidence into account; and even where the evidence suggests one thing, there is no way to exclude structural priming effects in those lists—after all, it is possible that the list was provided, or at least extended, due to chained activation of similar lexemes.

In spite of these limitations, we suggest that there are potential cases of both self-priming and other-priming by the prompt in Falko noun morphology. We chose transpositions as our example here for self-priming. In Figure 12, the author of text dew07_2007_09 produces a series of transpositions with a peak at the beginning and several recurrences of this morphological noun type throughout the whole text. This distribution fits well with the observation that priming effects decrease with increasing distance from the prime. A similar distribution can be seen in dcs007_2007_10, whereas dhw022_2007_06 and dhw015_2007_06 exhibit constant recurrences of transpositions.

The usage of non-native nouns in the university topic subcorpus of Falko is an example for other-priming. The prompt for these texts contains complex nouns of the neo-classical word formation type (labeled as non-native) and primes the usage of other nouns of the same type. This can be seen in the numerous peaks for non-native nouns in the same plot, texts fu081d_2007_10 and fu082d_2007_10. Crucially, the dispersion of peaks indicates that the effect is not due to mere listing of non-native words within a single window.

In the case of dcs007_2007_10, we also find a similar pattern to the Kobalt data, namely the clustering of a category type in one part of the text vs. another in another part, with transpositions peaking earlier in the text than non-native nouns.

Our results show clearly that a cumulative account even of individual texts still masks intra-individual, or procedural, variation that occurs in peaks that in several cases shift or alternate between categories. While it is in principle possible to analyze our syntactic categories in the same way, there are some stricter limitations to both the necessity and the clarity of the analysis. Since syntactic elements appear to converge to a higher degree between speakers, cumulative counts of those are less problematic at least methodologically—if speakers can be expected to level out across text even in texts of divergent length, this would imply they also level out in shorter spans, and hence cumulative counts are less misleading overall. At the same time, accounting for syntactic priming by category is theoretically more complicated. This is due to the same reason as stated above for noun morphology, namely their high similarity (every noun is a noun, and they tend to occur frequently—what could provide certainty that this is due to priming?) and coordination (some participants like to list activities of a similar kind, like “reading books, magazines, or the newspaper,” in which three accusative objects would occur within a very small window. Whether this should be considered priming is unclear.) The charm of morphological priming is that words can be diverse within the same morphological category. It appears less likely that a participant will intentionally reuse the same category or coordinate several words of the same morphological category, but not the same lexeme, in the same way as a syntactic construction would allow. We will not exclude that possibility, but we will leave it for future research.

## 5. Discussion

We began with the observation that L1-speaker data, aside from stratified or situational variation, is often conceptualized as a more homogeneous baseline in learner corpus studies, against which learner language is modeled as more diverse. While there has been a general paradigm shift in multilingualism research that models native speakers as less homogeneous than it used to, this paradigm shift is based on a prism refracting the formerly monolithic model of native language into a large number of diverse group memberships, not unlike the intersectional approach to society in general. For example, native speakers are not a homogeneous group if attributed as such by country of residence or exposure to the target language alone. They may differ by a number of language-external factors (such as age, region, or socio-economic status) and several language-peripheral factors (such as reading experience and linguistic aptitude), some of which may be explanatory in the diversity of use. There are also clearly influences of linguistic environments that trigger one linguistic realization over another, as if setting switches probabilistically and independent or only partially dependent on other characteristics of the speaker.

However, this is *still* a stratified view. We maintain that even approaches accounting for such systematic differences do not do justice to the full variability present in native speaker data. Quantitative and qualitative differences are strongly expressed even in the analysis of a highly homogeneous group of speakers, but this appears to be the case for some linguistic levels more than others. In other words, the speakers in our corpora were selected to be as homogeneous as possible, limiting participation to the literal same classroom in the case of Kobalt, yet *still* we find quantitative differences in morphology, but relative homogeneity in syntax. Both the high degree of variance in German morphology and the divergence between degrees of variance between linguistic levels is to our best knowledge previously undescribed.

We have further shown that all except the vanishingly rare categories are equally subject to high degrees of variability, even those that would be considered a prototype or baseline category such as simplex verbs or nouns; and that even relatively coarse aspects of the total distributions, such as the order of categories by frequency or even the category ranking highest by frequency, could not be determined across speakers in our corpora. This is in spite of highly controlled elicitation conditions as well as identical prompts between participants.

This is relevant in the context of a growing interest in morphological complexity in SLA. It is also highly relevant in the context of learner corpus and other usage-based studies, that largely work from a contrastive paradigm even if they do not explicitly state this, but as is evident from the methodology they apply.

Studies that concentrate more on the nature of presumed input of learners in most cases also do this in contrast to a native control group of some sort. Linford et al. (2016), where the make-up of the control group is one of the independent variables, do not only consider a global and local corpus as comparison, but entertain the possibility of a control group of other learners^23^. The crucial point, however, is that for none of the group-data—be it from the local or global corpora—inter-individual variability is reported. Geeslin et al. (2013) and some of the references therein are an exception insofar as they do report standard deviations of their control group. However, our data further shows that native speakers do not simply differ in their realization or non-realization of a binary category, but *in the whole composition* of their morphological subclasses for nouns and verbs, but *the same speakers* do *not* differ to a comparable degree in higher-order and more systemic syntactic compositions.

We have further shown that even the degree of intra-individual variation can be high and appears to follow systematic patterns organized by procedural effects. Variable degrees of intra-individual variation would be expected to transcend into variable degrees of inter-individual variation. If, for example, some participants prime themselves to the use of particle verbs, they will use them more overall than those who are less susceptible to self-priming or who do not happen to use a particle verb before they finish their text. This highlights the non-ergodicity, or path-dependence, of the writing process. However, in corpus linguistics, corpora are largely treated as static, non-dynamic data, with perhaps the exception of dialogue corpora and the smaller number of corpus-based priming studies that are available to date. Our data suggests that these aspects may deserve more attention in the future.

One of the reasons for why inter-individual differences of this scale even among (theoretically) homogeneous control groups have not attracted more attention so far may be in the syntactic and/or lexical focus of much of corpus research: Gurzynski-Weiss et al. (2018) have shown that the preference for a specific form of subject expression in L1-Spanish correlates with grammatical context and situational setting, while Linford et al. (2016) report distributional differences in context-integration between their learners and control groups. Since these contextual variables (level of attainment being one of them) lend themselves quite well to explaining the observed differences, there seems to be no need to delve deeper into inter-individual differences on the side of the control group. The morphological phenomenona observed in our data, on the other hand, evade the same kind of explanation. All of our speakers realize nearly all of the forms, and where they do not, it is clearly not a function of attainment.

In conclusion, various usage-based models think of L1 frequencies as representations of relevant quantitative properties of the target language, and frequently interpret L2 frequencies as over- or underuse. If subclasses are not equally distributed across native speakers, like our data shows for verb and noun morphology, this perspective needs to be expanded to include inter-individual and perhaps even intra-individual differences in L1. We will briefly discuss methodological implications for learner corpus studies and theoretical issues that arise for cognitive/usage-based models of SLA.

### 5.1. Methodological Implications

We began this paper by stating that in many learner corpus studies, native speaker data is used as a control group for comparison with learners. In the contrastive paradigm, higher or lower frequency of occurrence of various linguistic elements in learner data is frequently viewed as evidence for a learner's target language competence. This methodologically implies native speakers as somewhat idealized carriers of the target language that converge both qualitatively and quantitatively, even where the research paradigm theoretically states otherwise. Learners are naturally presumed to exhibit higher variability, as are bilinguals in general (Seton and Schmid, 2016, 341).

This does not match with our data of a (theoretically) very homogeneous group of native speakers of German. We conclude that it is therefore important to refrain from comparing groups by cross-corpus means without further investigation of variance and distribution, and we should not presume native speaker homogeneity *across linguistic categories*. For a valid group comparison, the distribution within the group must be both (a) known and (b) comparable. We cannot rely on median or mean values as long as the variability tendency of the phenomenon at hand is unclear, which means that for any corpus statistic, the inter-individual comparison *must* be accounted for and reported. This can complicate matters, especially where individual contributions are not trivially attributable or where the research questions requires the consideration of rare phenomena that do not always manifest in the writing of every individual. However, a quantitative analysis is only meaningful if we understand the underlying, expected, and measured distribution adequately.

This is especially relevant where statistical models are employed, because those typically rely on certain assumptions that may not be met by vastly variable within-group distributions. The most basic assumption of statistical models is that phenomena have a probability, which in frequentist statistics is defined as the outcome of each factor in terms of relative frequency of an infinite series of random experiments. In other words, if I draw samples from the same population a large number of times, over time, the relative frequency for each state (each morphological subclass, for example) should stabilize, i.e., converge to an idealized value, which is the probability. If it does not, this can be due to the phenomenon not having a stable probability: it may be too dynamic, e.g., driven by intention, the invisible hand of cognitive and procedural factors such as priming, or a combination of those two with more general frequency patterns. In that case it might best be understood as a complex dynamic subsystem (individual grammar/*parole*) within a larger complex dynamic system (speaker group language/*langue*)^24^. If a phenomenon does not have a stable probability, statistically inferring from a sample to a population is meaningless (see Shadrova, ress, for a more in-depth argument).

In our data, speakers do not converge to one another in their use of more fine-grained categories in a single text, while they do appear to converge (within a range) in some other categories. Would more data resolve the issue? Do speakers converge to one another, i.e., follow general frequency patterns in the use of subclasses of verbs and nouns, but a single text does not provide a sufficiently large speaker-specific sample? Do they follow different, but contextually stable frequency distributions, for example by text type or register, and would these converge between speakers? Do they not converge to one another, but stabilize in their own frequency patterns—i.e., are there idiosyncratic frequency distributions for each speaker? Or is there simply no convergence between or within speakers, i.e., should we allow for random fluctuation within a range of between 15 and 66% of simplex verbs in Kobalt (DEU_001 vs. DEU_011) and 0 and 53% of modal verbs (DEU_018 vs. DEU_005)? In this case, we would have to accept that a simplistic groupwise comparison of the phenomenon based on frequencies is pointless. Statistics is a scientific belief management system designed to filter the signal from a noisy (variable) environment. However, measurements that hit both floor (0 occurrences) and what could be considered ceiling (53% modal verbs) complicate the analysis. It is possible that looking more into the shapes of the distributions and their interactions with other category distributions would yield clearer results. Either way, if native speakers, i.e., target language carriers, use between zero and as many modal verbs as reasonably possible, the precise mapping and comparison of learner data to this raises methodological questions.

More importantly, our results raise linguistic questions: what is going on in the language of speakers that do not use any modal verbs? How do they construct modality instead? What is different in the language of speakers who barely use auxiliaries, copula, or constructional verbs, but many modal verbs? How does their language differ from all the other speakers in the corpus? Should we attempt to capture morphosyntactic speaker profiles instead of individual varieties? These questions in turn trigger methodological considerations that go beyond the question of adequate statistical description and analysis.

### 5.2. The Role of Frequency in Usage-Based Accounts

As has been briefly discussed in section 2, usage-based accounts of language acquisition and production make a strong point of emphasizing the role of frequency in the input. This applies to the whole range of the continuum from syntactic constructions to individual words and word co-occurrences (Bybee and Hopper, 2001; Gries and Wulff, 2005; Ellis and Frey, 2009; Ellis, 2012; Goldberg, 2013; Diessel and Hilpert, 2016; Hilpert, 2017; Gries, 2019, and many others). The idea is that speakers are sensitive to frequency distributions because frequencies of linguistic elements acquire neuronal correlates by means of entrenchment (strengthening of neuronal pathways through repetition, resulting in effortless reproduction of the entrenched frequencies). An element that is frequently heard or seen will be frequently produced and more easily recognized. They also make the case that all linguistic units exist on a continuum of form-meaning pairs that in principle are learned in the same way, or that “it's constructions all the way everywhere” (Boogaart et al., 2014, 1).

Our data provides challenges to this account. It has been collected from participants from homogeneous backgrounds – to the extent that our high school students would be faced with similar books at school, share significant amounts of daily conversation and a similar social environment in many ways. Still, they either do not arrive at the same distributional abstractions, or do not reproduce those abstractions in the same way. This means that either (a) frequency in entrenchment is not automatically mirrored in production, (b) that there is another factor determining frequency distributions that is currently being overlooked (such as latent register differences between texts) or simply (c) that not all constructions are entrenched with frequency. But what would that imply for “constructions all the way down” (Goldberg, 2006)?

In our data, we do not find the same divergence between individuals for some higher-level syntactic relations, parts of speech and dependencies. It is possible that this is not an effect of abstraction/concreteness, but one of relational function: unlike verbs or nouns of different morphological types, the different dependency types or parts of speech form a system. Thus the total 100% of all dependencies in a text are mutually interdependent to a large degree—one can often not easily add a verb without also adding nouns, or a noun without also adding a determiner/quantifier etc^25^.—while the elements tallied in the other categories are mutually independent (using an extra prefix verb does not grammatically enforce the next particle verb, for example). A system is defined by the mutual interrelationships of its elements (Mesarovic, 1964), producing a latent structure which might be accountable for stable frequencies. It is possible that speakers are not as much sensitive to *frequencies* as they are to *proportions* within a (sub-)system, or in other words that frequency is an epiphenomenon of structured inventories of signs, not a feature of the signs themselves^26^. This would go against the idea of equality of all linguistic signs and categorizations as it is prominent in usage-based accounts (“constructions all the way down” Goldberg, 2006, 18). For a valid quantitative statement, one would then need to define the respective subsystem first.

One relevant question in this regard is whether the differences in morphological category distributions could be explained by looking at lexical, rather than morphological, frequencies. Theoretically speaking, morphologically complex words could in principle be realized without taking note of their complexity (as chunks or words without deeper analysis). While it is necessary to have an abstraction over forms for felicitous productivity, this is not necessary for the plain use of form. One could argue that it is possible that complex verb forms go largely unanalyzed in some or most speakers—that they are fully lexicalized and their distributions merely an epiphenomenon, that “meaning overrides frequency” (Jolsavi et al., 2013). However, as is frequently argued in usage-based approaches, schemas must be accessible in lexicalized forms, too, since productivity and generativity is considered to emerge from usage, and grammar from the use of lexemes (Booij, 2013; Zeldes, 2013; Hilpert, 2019, and others). If the schema is present in all use, and frequency is part of the schema, would we not expect less variable distributions between speakers?

With respect to the the data model and analysis, if word frequencies were stable, so would be morphological frequencies, because words do not change their mophological class. A higher level of abstraction would always reduce noise due to the loss of individuality of the lexemes. If anything, morphological categorization should level out the variance (higher dispersion) from more granular categories such as lexemes. There is also no evidence for lexical convergence or considerable overlap between authors in our corpora.

The problems with this perspective run deeper, though. Statistical approaches to word frequencies as quantifications of the lexicon in use have a long history in corpus linguistics (Baayen, 2002; Stefanowitsch and Gries, 2003; Gries and Wulff, 2005; Gries, 2013, 2019; Brezina et al., 2015, and many others). However, there are major mathematical and philosophical flaws. If word frequencies are not stable, i.e., stationary, and ergodic, i.e., path-independent (unaffected by factors such as priming or intention), they cannot be validly used for statistical computation. This is because all frequentist statistics relies on the central limit theorem, which does not hold true in systems that are non-ergodic or not stationary (Shadrova, ress; Schmid, 2010; Koplenig, 2017). There is mathematical research suggesting that language is overall non-ergodic (Dȩbowski, 2018). This could potentially be tackled by defining ergodic subsets. However, there is also evidence that even large corpora may not be stationary (Piantadosi, 2014; Shadrova, 2020) shows that for Kobalt, there is barely any lexical overlap between texts.

Most importantly, however, the way words are distributed in natural language makes word frequencies largely an artifact of corpus size. While there are groups of words that tend to occur more frequently, highly frequently, and so on, they escape any precise or meaningful quantitative categorization. Words as they occur in corpora follow a long-tailed distribution which is marked by a few highly frequent and some less frequent words, and a very large number of words that occur only once (*hapax legomena*). The larger the corpus, the more hapaxes. This is true of individual text and text corpora equally. For most words, their frequency thus is 1 divided by corpus size. There is no evidence that word frequencies are stable (stationary) in any corpus size. If it were, there could be no productivity, because all new words would take up space. It is clear that word frequencies can fluctuate more systematically (some disappear; some disappear, then reappear), however, such fluctuations are unpredictable beforehand. It is the statistical equivalent to rolling a die with a changing number of sides. The same is not true of morphological categories, which at least synchronically show some stability and a level of certainty of occurrence. While not every one of our participants uses all morphological categories, most classes are well represented and pooling only a few texts leads to good coverage of all classes. The same is far from true for lexemes in *any* corpus size.

It is of course possible that other factors can explain the divergence in individual distributions of classes of verbs and nouns in native speaker writing in these corpora. It might be a matter of aptitude or experience, style, or cognitive biases such as priming. Even then, usage-based linguistics needs to clarify the role of frequency and variance across linguistic categories in interaction with these factors. This is necessary for descriptive adequacy—if we observe heterogeneity in frequency realizations between native speakers, our theoretical models should capture this fact. It is equally necessary for explanatory adequacy—something makes speakers arrive at different frequency realizations in some, but not all categories, and usage-based theory at present does not provide a mechanism for this.

The divergence between category frequencies in production is also relevant for the question of input. Since the data we collected is semi-naturalistic—it has been collected for a linguistic purpose, but it is not unlike tasks that students are faced with in high school or college in Germany—we can assume that this is a realistic production scenario. If it is a realistic production scenario, it must also be a realistic input scenario: if speakers can choose to use simplex verbs between 15 and 66% in a text, then those who read those texts are equally confronted with such differences. While in a corpus, frequency may or may not level out, speakers outside of corpus linguistics are rarely confronted with a corpus to read. How are speakers not confused in their entrenchment of the frequency of morphosemantic constructions such as “particle verb” or “simplex verb” if those frequencies fluctuate by such vast amounts between texts? What does it say about a phenomenon if it allows for high degrees of seemingly random fluctuation?

We will not exclude the possibility that there is some stratified variation between speakers in our corpus that we have not been able to account for yet. Wherever data occurs with high variation, the possibility of subgroups, such as a speaker typology by preference or style of expression, should be considered. This remains for future research and modeling. For this analysis, we chose to look into more procedural factors, which tend to be less in focus in corpus linguistic research. Our analysis is consistent with a priming-based explanation of at least some of the variability in our corpus. If the occurrence of one particle verb primes for three or four more such verbs, this would have great impact on the overall distribution in a text of 600 tokens, for example. It is plausible to assume that we find less variation in the more global syntactic phenomena due to varying degrees of susceptibility to priming. Global syntactic categories may be largely fixed through inherent constraints of the system, while morphological and other more fine-grained categories may be more susceptible to priming. Yet others may be subject to more free choice or control through speaker intention, resulting in stylistic choices. Such effects may differ by various factors, such as speaker aptitude, writing experience, or different register perception and knowledge.

Of course, this is a slippery slope. It might be tempting to suggest that fluctuations in frequency, whether they stem from preferences or priming, are a “performance” issue similar to how traditional generative grammar has declared ungrammatical sentences out of scope of syntactic research. This would miss out on a chance to learn about deeper structural differences between those categories that allow for fluctuations vs. those that do not appear to do so, which has multiple repercussions on procedural (connectionist) theories of language learning, production, and productivity. It would also pose challenges to the development of more adequate models for prediction and analysis of results in quantitative corpus studies. Most importantly, it would introduce a major inconsistency into constructionist models of language acquisition, because it would define frequency as both *relevant in acquisition and reception* and *irrelevant in production*, which is logically inconsistent, since reception depends on production.

We would like to emphasize that none of this is to say that there are no differences between L1 and L2 usage of morphological categories, or that “everything is just very, very diverse and cannot be captured”. Rather, we argue for precise modeling from factors already available in many corpora, namely a document-wise analysis and consideration of a view of text as process. Native speaker writing is more complex than is frequently accounted for at present, and a more comprehensive view would emerge from an adequate representation of methodological decisions in theoretical modeling as well as vice versa.

### 5.3. Conclusion and Future Research

In this paper, we have presented data from two task-specific German L1 corpora that were initially collected as control corpora for second language acquisition studies. We have shown that in these two corpora, which are carefully compiled and controlled by a number of factors such as text type, writing conditions, participant background, and prompt, native speakers show high quantitative variance in the distribution of morphological subclasses of verbs and nouns, both between and within speakers. We have also shown that part-of-speech and syntactic dependency distributions do not appear to be subject to the same variability. As our morphological data suggests, it appears that even the gratifying departure from the assumption of native speaker homogeneity as it is represented in variationist and multilingualism-centered perspectives is not yet taking things far enough.

Future research needs to clarify the stability of the degrees of variance we find in native speaker writing for different levels of linguistic description. Do speakers, for example, show stable and persistent individual distributions of morphological types in verbs and nouns, or is high variance triggered through priming? How much of this is driven by intention/rhetorics, and how much is cognitively biased? What is the role of speech rhythm/accent patterns and phonetic priming, and what is the role of semantic priming in the repetition of (seemingly) abstract structures?

Our results highlight the importance of accounting for inter- and even intra-individual variance in corpus studies. In fact, some phenomena show such high degrees of variance that a quantitative comparison without further specification of the model appears pointless. This is crucial for quantitative studies—in order to study differences between language learners and native speakers, we need to know which phenomena allow for a meaningful quantitative comparison and which ones do not. Beyond this empirical implication, theoretical questions arise with respect to the role of frequency and item distributions that have traditionally been emphasized in usage-based linguistic theory, both in language learning and production in L1 and L2. If speakers produce vastly different quantitative outputs, then the role of quantitative entrenchment and its repercussions on language in use becomes much less clear and its centrality and implication as a lever in language learning may need to be reassessed at least for some linguistic levels.

Finally, if syntactic units are easy to quantify and converge quickly, while morphological units show different behaviors, and lexical material is even more difficult to grasp in mathematically valid ways, the idea of “constructions all the way down” (or “all the way everywhere”) should be discussed in a more differentiated manner. While all these linguistic elements can be conceptualized as signs or form-meaning pairs on some level, the mechanisms facilitating their acquisition and production appear to differ at least with respect to their sensitivity to frequency and their (in)equation of frequency and entrenchment.

## Data Availability Statement

The datasets presented in this study can be found in online repositories. The names of the repository/repositories and accession number(s) can be found below: https://www.zenodo.org/record/3584091; 10.5281/zenodo.4752308.

## Ethics Statement

Ethical review and approval was not required for the study on human participants in accordance with the local legislation and institutional requirements. Written informed consent from the participants' legal guardian/next of kin was not required to participate in this study in accordance with the national legislation and the institutional requirements.

## Author Contributions

All authors listed have made a substantial, direct and intellectual contribution to the work, and approved it for publication.

## Funding

Research Unit Emerging Grammars in Language Contact Situations: A Comparative Approach, FOR 2537, SH 1685/1-1, LU 856/16-1, 313607803, (AS, AL, and PL). SFB 1412 Register, funded by Deutsche Forschungsgemeinschaft, 416591334 (JL and AL). Crosslingual Language Varieties, funded by Deutsche Forschungsgemeinschaft, LU 856/13-1, 398186468 (SS and AL).

## Conflict of Interest

The authors declare that the research was conducted in the absence of any commercial or financial relationships that could be construed as a potential conflict of interest.

## Publisher's Note

All claims expressed in this article are solely those of the authors and do not necessarily represent those of their affiliated organizations, or those of the publisher, the editors and the reviewers. Any product that may be evaluated in this article, or claim that may be made by its manufacturer, is not guaranteed or endorsed by the publisher.

## Footnotes

^1^One notable exception is Mulder and Hulstijn (2011).

^2^The data is described in detail in section 3. Annotation details are provided in the Appendix in Supplementary Materials.

^3^One could argue that it is next to impossible to keep the genre, register, or broader function of text produced under elicitation homogeneous (Shadrova, 2020; Lüdeling et al., 2021; Wan, 2021). In our case, the high school students prompted for Kobalt have all learned to produce the type of argumentative text prompted for our corpora in the very same classroom over years. While this does not mean they all attempted the same register or genre in production, it is plausible to assume that their acquisition background is very similar in that respect.

^4^The desirability of the latter is relativized by Birdsong and Gertken (2013, p.118) insofar, as “knowing more about the nature of natives' linguistic system is of theoretical significance in its own right”.

^5^Generative grammar perspectives on individual differences, the role of input, and ultimate attainment can be found in Cook (1991), Borer (1996), Cook (1991), Hilles (1991), White and Genesee (1996), Yang (2004), Rothman and Iverson (2008), Rothman and Iverson (2008), and White (2015).

^6^There is of course more to say about the similarities and differences between usage-based and nativist language acquisition theories. One could argue that the introduction of the concept of *learned attention*—blocking of certain categories, constructions, etc. by means of the learner's L1 for acquisition of L2-categories and constructions—is rather close to the idea of an innate principles and parameters already being set (and thus, in a similar way, “occupied”) by the L1, as in generativist approaches. Similarly, the covertness of entrenchments, and the limitations to their access, resembles the hiddenness of UG's *competence*. The predictions of usage-based accounts about what a learner opposite a native speaker knows can still be different from generativist accounts, even given the same data. Since in a usage-based account performance and its distribution is seen as direct expression of the underlying entrenchment, the same performance by a learner and a native speaker would result in assuming that their categories, words, and constructions are entrenched in a similar way. A generativist account first of all might not consider the data valid for taking hold of the underlying competence in either of the two, and secondly, might not conclude that similarity in performance means similarity in competence.

^7^Some studies that explicitly take into account variance in the input that learners are exposed to do so on the basis of group characteristics. Conceptually, these studies model differences between the *input* for learners and non-native speakers. This is unlike the central question of early SLA research, that often had a more deficit-oriented perspective, and asked to what extent the *output* of learners conforms to that of native speakers (for a critical review, see for instance Klein, 1998). A method to determine both is the comparison between corpora. Notably, even despite not explicitly stating any adherence to Contrastive Interlanguage Analysis (CIA), studies under a variationist umbrella that use naturalistic data often make very similar methodological decisions in comparing their corpora. In fact, Eskildsen and Cadierno (2015) describe the different foci of linguistic patterns vs. sociolinguistic classifications as cognitive/usage-based (CUB-SLA) theories on the one hand, and theories based on conversation analysis (CA-SLA) on the other, and view them as complementary rather than mutually exclusive.

^8^A problematic aspect of this study is the definition of “frequent” vs. “infrequent”, which is pragmatically—and understandably—drawn arbitrarily at the 1% threshold of verb tokens in the corpus. However, this will necessarily massively fluctuate with corpus size and type. Where theoretical conclusions from frequency are drawn, the set of affected words should be stable, but derived in this way, it cannot be stable.

^9^For critical stances toward lexical statistics (see Shadrova, ress; Kilgarriff, 2005; Schmid, 2010; Koplenig, 2017).

^10^This has also been discussed for one of the corpora used in this study, Kobalt, in Shadrova (2020, ch. 7).

^11^After primary school (year 4 or 6, depending on the federal state), students are divided into three general tiers, so-called *Haupt-* and *Realschule* and *Gymnasium*. *Haupt-* and *Realschule* end after year 10 and aim to prepare students for vocational training, which is accompanied by ongoing education at professional school *Berufsschule*. Students completing their studies with the high school degree *Abitur* at *Gymnasium* acquire the right to study at a university or college. The separation of students into tiers attracts much critical debate for being known to be a highly socially selective procedure restricting upward mobility. Some schools offer integrated schooling (*Gesamtschule*), but they still follow the principle of separate degrees, and students are usually taught separately by attempted degree in several subjects. Options to enter university without *Abitur* are very limited, especially outside of medical or engineering subjects, from which we did not collect data. In Germany, only 21% of the children of parents without academic degrees begin college studies, while 74% of parents with academic degrees do. Ratios are even more contrasted in the humanities, and also locally, since universities use cut-off marks based on student's *Abitur* grades to limit admissions, which affects Berlin in particular. *Abitur grades* are further known to correlate with socio-economic status to a lamentable degree.

^12^Since manual annotation is laborious and resource-intensive, we refrain from adding the complementary annotation layers to the respective other corpus, although, obviously, nouns were also used in Kobalt and verbs in Falko. Since we do not attempt a direct comparison between the two corpora, this should not constitute a problem.

^13^We exclude the category *gehen*_*cx* from our analysis. It labels the verb *gehen* in the constructional use of *Wie geht es dir?* ‘how are you doing?’ and is used at deceptively high rates since it is part of the prompt. We believe it cannot be considered well in the analysis of priming either, because it is highly salient in this context and is used with clear intention for text structuring purposes. We believe it should thus not be compared with the other categories.

^14^Some complex verbs in German are not analyzed in the same way by all speakers. This is especially the case for complex verbs that incorporate nouns, like *staubsaugen*, as opposed to the phrasal variant *Staub saugen* (‘to vacuum’, literally ‘to suck dust’). Some speakers read it as a prefix verb, *er staubsaugt, ich habe gestaubsaugt* (‘he is vacuuming’, ‘I have vacuumed’, literally ‘he dustsucked’, ‘I have dustsucked’)), while others read it as a phrasal unit (*er saugt Staub, ich habe Staub gesaugt*, literally ‘he sucks dust’, ‘I have sucked dust’), which then lends itself to a particle verb analysis: *er saugt staub, ich habe staubgesaugt*. Similar patterns can be observed in newer verbs like *downloaden* (‘to download’), *um das Video downzuloaden* (particle) ‘in order to download the video’ vs. *um das Video zu downloaden* (prefix). In Kobalt, there were only very few cases of this type, and they were analyzed as closely as possible to the original writing. If a participant wrote them as phrasal units, they would be analyzed as a simplex or a support verb, depending on the compositionality of the combination. If a participant wrote them as one word, they were analyzed as prefix verbs, unless there was syntactic evidence for the separability of a particle, as in *downzuloaden*. Often, speakers avoid commitment to one analysis by using syncretic forms, for instance *Man kann das Video downloaden, indem man auf den Link klickt* (‘One may download the video by clicking the link’, prefix or particle), rather than *Um das Video downzuloaden (particle)/zu downloaden (prefix), muss man auf den Link klicken* ('In order to download the video, one has to klick the link').

^15^In some cases the connection is synchronically fairly far removed, as in *raten* ‘to guess, to advise’ and *verraten* ‘to betray’. A more detailed discussion can be found in a comparative study of complex verb productivity in German L1 vs. L2 in Lüdeling et al. (2017).

^16^This would matter in an actual contrastive scenario, because it can provide insights into the structure of the morphological system, for example whether morphologically complex verbs take an equally central position among the other subclasses in L2 and L1, and answer questions concerning productivity and lexicalization, complexity, or the development of aspect and perspectivation through verb modification. Lower morphological complexity as an L2 phenomenon has generally gained interest in the SLA literature in recent years (Zeldes, 2013; Ehret and Szmrecsanyi, 2016; Lüdeling et al., 2017; Yoon, 2017; Brezina and Pallotti, 2019; De Clercq and Housen, 2019, and others).

^17^We are aware that pie charts are not an ideal type of data visualization for most purposes, because they tend to make a comparison of exact proportions difficult. This is due to limitations of the human mind, that seems to be less well-equipped to compare and interpret dimensions from angles other than 90 degrees. However, in our case, we will compare a large number of compositions, i.e., distributions of several (more than three) factors. This becomes very difficult to read in stacked bar plots, since two or three factors can be ordered by relative size of each factor, but four cannot, making the bars and colors very noisy in perception. Pie charts have shown to be the most efficient at visualizing the differences both *between factors* and *between texts* in easily graspable ways. The central point is the distribution of subclasses within each category, i.e., whether the pies look similar or different in their division into pieces. If subclasses of categories were similarly distributed, all pies should be cut in similar ways, i.e., have pieces of similar shape and size, as is the case in some of the later plots in this paper. For the morphological subclasses, they tend to not be, and that is the point. Precise percentages or individual mappings do not matter much and will only be referred to for exemplification.

^18^German has six: *wollen, können, sollen, müssen, dürfen, mögen/möchten*, ‘to want’,‘to be able to’, ‘to be obligated; shall; epistemic must’,‘deontic must, to have to’, ‘to be allowed to’, ‘would like to’).

^19^More individual distribution plots are available through a Zenodo repository, 10.5281/zenodo.4752308. Only three out of four topics are available with corrected dependency labels in the current version of Falko, hence we are unable to show distributions for the *university* topic at present.

^20^There are too many plots to present legibly in this paper. All remaining plots can be found along with the scripts for analysis in a Zenodo repository under 10.5281/zenodo.4752308.

^21^The number of tokens for each window is chosen arbitrarily. Since the windows overlap, no information is technically lost in smaller or larger windows. However, if the windows become too large, two peaks can be bridged, suggesting ongoing activation where in fact, there is a slump. If the windows are too small, peaks never reach levels higher than two or three, potentially clouding existing activation. A more exact calibration of this measure remains for future research and should be conducted in alignment with psycholinguistic research.

^22^We did not account for repetition across the whole text, but plan to do so in future research.

^23^While this is quite plausible, the way it is operationalized is problematic: the learner data taken as verum group is simply added to the native bilinguals' group data that already serves as one of the control groups, and this “supergroup” is then entered as the third control group, albeit neither independent from the learners nor the native bilinguals.

^24^For *langue* and *parole*, see Saussure ([1916]1983). For the modeling of language as a complex dynamic system (see Ellis, 2006; Five Graces Group et al., 2009; Lowie and Verspoor, 2019), among others.

^25^Except in the coordination of lists of activities, as in *they drank tea and danced and laughed*. However, even this will sooner or later trigger nouns: …*and played guitar and told stories…*, and also cannot be continued ad infinitum in a realistic context due to limitations in processing as well as its communicative pointlessness.

^26^A similar suggestion with respect to coselectional constraints on verb-argument structures has been made by Shadrova (2020, 264-265).
