# Supplementary material for: A Challenge for Contrastive L1/L2 Corpus Studies: Large Inter- and Intra-Individual Variation Across Morphological, but Not Global Syntactic Categories in Task-Based Corpus Data of a Homogeneous L1 German Group
Source: Front Psychol. 2021 Nov 25;12:716485. doi: 10.3389/fpsyg.2021.716485 (PMC8661037; doi:10.3389/fpsyg.2021.716485)
Supplement: Supplementary file 1 [file Data_Sheet_1.pdf]

frontiers

Supplementary Material

APPENDIX, SUPPLEMENTARY TABLES AND FIGURES

Classification of verbs in Kobalt

Verbs in Kobalt were classified in the following way:

- Syntactic verb categories: Auxiliary, copula, modal, modifying, and constructional verbs:
  - **Auxiliary verbs (labeled *aux*)** are functional verbs used to construct tenses and passive voice, namely *haben* ‘to have’, *sein* ‘to be’ and *werden* ‘will’,‘to be (+ passive)’.
  - **Copula verbs (labeled *copula*)** are functional verbs used to link subjects to predicates, mainly *sein* ‘to be’.
  - **Modal verbs (labeled *modal*)** are functional verbs used to syntactically construct modal constructions such as *wollen* ‘to want’, *können* ‘can, be able to, may’, or *müssen* ‘must, to have to’ in the sentence *Wir wollen/können/müssen nächste Woche freinehmen* ‘We want to/can/must take next week off of work’.
  - **Modifying verbs (labeled *modifying*)** are similar to modal verbs, but trigger a full infinitive with *zu* ‘to’, as in *Du* ***scheinst*** *das nicht* ***zu mögen*** ‘you do not seem to like that’.
  - **Constructional verbs (labeled *cx*)** are verbs used in certain syntactic constructions such as infinitive (*Es ist viel zu tun* ‘there is much to do’) or reflexive constructions (*Er lässt sich das nicht länger gefallen* ‘he will no longer take it’, literally ‘he does no longer let himself accept/like it’).
- Morphosemantic categories of lexical verbs: Simplex, particle, prefix, and support verbs:
  - **Simplex verbs (labeled *simplex*)** are verbs with full lexical meaning and morphologically basic structure, such as *schreiben* ‘to write’ or *glauben* ‘to believe’. This category includes the lexical use of ambiguous verbs such as *haben* ‘to have’ as in *Er hat ein Auto* ‘He has a car’, whereas *haben* in its auxiliary function is classified as *aux*; and does not include the constructional use of lexical verbs, since those are classified as *cx*.
  - **Particle verbs (labeled *particle*)** are complex German verbs that, similarly to phrasal verbs in English, compound a simplex or complex stem with a particle, for instance: simplex verb *lesen* ‘to read’, particle verb *vorlesen* ‘to read out loud’. Unlike in English, the particle occurs as a morphological part of the verb itself, but is split in certain syntactic environments: *Er will ihr die Geschichte* ***vorlesen*** ‘He wants to read the story out to her’, but *Er* ***liest*** *ihr die Geschichte* ***vor*** ‘He is reading the story out to her’.
  - **Prefix verbs (labeled *prefix*)** are composed of a simplex stem and a prefix, such as *verbiegen* ‘to bend out of shape’ vs. *biegen* ‘to bend’, or *verschenken* ‘to give away as a gift’ vs. *schenken* ‘to gift’. Unlike particle verbs, prefix verbs are never split.
  - **Support verbs (labeled *support*)** are part of support verb constructions (*Funktionsverbgefüge*) and form non-compositional meanings in phrasal use with a noun component, for example *eine Rolle spielen* ‘to play a role’ or *zur Verfügung stellen* ‘to provide’, literally ‘to put at disposition’. Since this is a more fuzzy-edged category, support verbs were annotated minimally, i.e. only for the more evident cases. Support verbs tend to be morphologically simple, however, in some cases, they can include prefix or particle verbs (6 out of 34 lexemes in Kobalt, 5 of them hapaxes, one occurring twice, i.e. 6 out of 93 total occurrences in the corpus). For the largest part, a failure to recognize a

support verb, or a positive miscategorization as one, would shift proportions between simplex and support verbs.

Morphological categorization of complex nouns in Falko

- **Determinative compounds (labeled *kdet*)** are combinations of two free morphemes such as *Hausdach* ‘roof of a house’ where the head *dach* carries the central semantic information and the non-head *Haus* specifies the concept as a roof belonging to a house.
- **Phrasal compounds (kphras)** are combinations of a head noun and phrases of variable complexity, cf. *Schönes-Wochenende-Ticket* ’Nice-weekend-ticket’.
- **Derivations (labeled *der*)** combine a free morpheme with a nominalisation affix like *-er* or *-ung* in *Wecker* ‘alarm clock’ (*weck(en)* means ‘wake sb. up’, *-er* is a agentivizer or instrumentalizer affix) or *Gleichung* ‘equation’ (*gleich(en)* means ‘equal, be alike’, *-ung* is a process or result nominalizer affix).
- **Conversions (labeled *kon*)** (here) are nouns like *Sitz* that are related to stems in a another syntactic category without overt marking of any kind, cf. the verbal stem *sitz* ‘to sit’. The category is assigned disregarding the question which category is the original one for the specific pair.
- **Transpositions (labeled *trans*)** are nouns derived from inflected words in other syntactic categories, like nominalised infinitives (*(das) Leben* ‘life’), nominalised inflected adjectives (*(die) Grünen* ‘(the) greens’) or nominalised participles (*(ein) Erwachsener* ‘(an) adult’).
- **Other nominalisations (labeled *nom*)** include nouns that do not pertain to one of the aforementioned noun classes but are visibly related to a stem in another category. This group contains nominalisations with the affixes *-t*, *-e* or *-st* that are non-productive and synchronically non-transparent. Furthermore, nouns derived from another category by changing parts of the stem or by adding circumfixes (mainly *Ge-...-e*) are subsumed under this class.
- **Simplex (labeled *sim*)** nouns do not involve any visible word formation structure, e.g. *Hand* ‘hand’ or *Tisch* ‘table’.
- **Non-native nouns (labeled *nnat*)** are nouns that contain a non-native stem and do not involve a native word-formation process on the highest level of morphological decomposition, cf. *Fakt* ‘fact’. They might, however, be the result of non-native word formation processes, cf. *Feminismus* ‘feminism’ or *Situation* ‘situation’.[1]
- **Abbreviations (labeled *kurz*)** include all types of processes leading to a shorter version of a word, such as clipping (*Uni* for *Universität* ‘university’), or abbreviations (*B.* for *Beispiel* ‘example’).

Randomized sampling to account for text length

In Falko, separate linear regression models for the proportion of each subclass depending on topic and text length yield high correlations for almost all topics, but only determinative compound and simplex noun proportions also significantly correlate with text length (see tables S1 – S3). Total occurrences of each noun class in individual texts are highly dependent on text length. This is a foreseeable artifact of high variance in text length (up to factor 9.5). Here, we draw noun samples of equal size from the corpus at random (scrambling the tokens across texts), we find that the measured variance for the occurrence of the vast majority of subclasses is indeed much higher than randomly expected. This is visualized in fig. .

In Kobalt, we find less strongly expressed differences in variance in equal-sized samples of 50 verbs (fig. S2). However, the general pattern still persists, as we can show from another perspective. For this, we sample expected variance under a presumed random distribution. We show this exemplarily for lexical verb

[1] Non-native nouns were categorized as to their origin and morphological structure on separate annotation layers. Details can be found in **?**.

subclasses in Kobalt. For fig. S3, we randomly sampled tokens without replacement from all of Kobalt based on the original text length distribution in our data. At first glance, the plot looks rather similar to fig. 2, although we do find some differences: expected variance in L1 is lower than measured for simplex verbs and prefix verbs (measured 0.011, expected 0.005 for simplex; measured 0.0039, expected 0.0023 for prefix verbs), but higher for particle verbs (measured 0.0035, expected 0.004). Support verb variance is almost as expected (measured 0.00149, expected 0.00146).

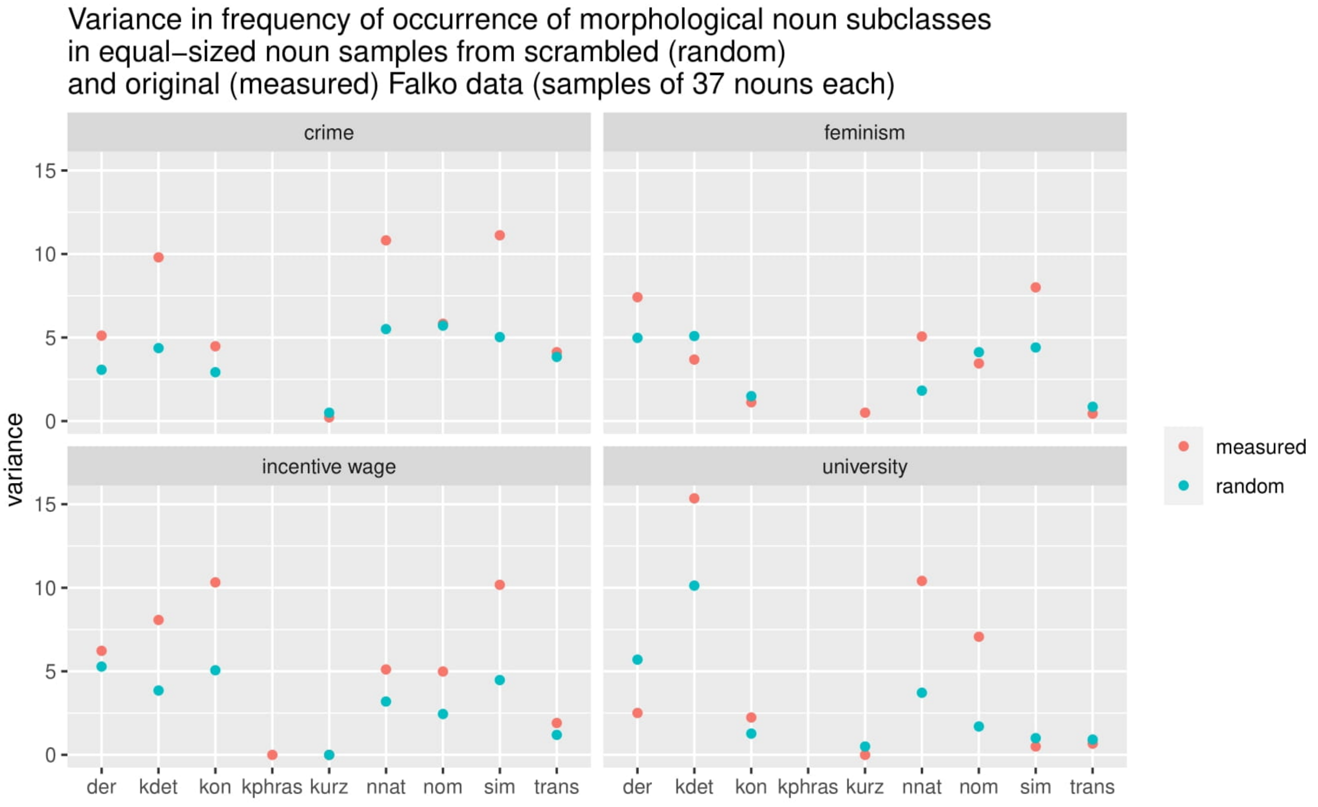

Figure S1. Variance in the frequency of occurrence in cross-corpus randomized and original (text-dependent) Falko data. The somewhat idiosyncratic value of 37 nouns per sample was chosen in order to include all texts (minimal number of nouns is 37).

Text length statistics

Kobalt: multiple R^2 = 0.1419 for prefix, 0.00037 for particle, 0.23 for support, and 0.5898 for simplex verbs. Kobalt is not divided by topic.

Falko: We do not report statistics for abbreviations (*kurz*) and phrasal compounds(*kphras*) due to low frequency of occurrence.

Table S1. Statistics for proportion of noun subclasses in Falko depending on text length and topic. Significant values for text length dependency are marked in red.

	Dependent variable: proportion of		
	derivations	determinative compounds	conversions
text length	−0.00002	0.041**	0.00001
	(0.00002)	(0.00002)	(0.00001)
topic: feminism	0.029**	−0.032*	−0.012
	(0.014)	(0.016)	(0.011)
topic: incentive wage	0.084***	−0.018	0.060***
	(0.011)	(0.014)	(0.009)
topic: university	0.041**	0.104***	−0.018
	(0.018)	(0.021)	(0.014)
Constant	0.130***	0.216***	0.081***
	(0.014)	(0.016)	(0.011)
Observations	94	95	95
R^2	0.387	0.329	0.406
Adjusted R^2	0.360	0.300	0.380
Residual Std. Error	0.046 (df = 89)	0.055 (df = 90)	0.037 (df = 90)
F Statistic	14.050*** (df = 4; 89)	11.050*** (df = 4; 90)	15.400*** (df = 4; 90)
Note:			*p<0.1; **p<0.05; ***p<0.01

Table S2. Continued statistics for proportion of subclasses depending on text length and topic. Significant values for text length dependency are marked in red.

	Dependent variable: proportion of	
	non-native nouns	nominalizations
text length	−0.00002	−0.00000
	(0.00002)	(0.00002))
topic: feminism	−0.012	−0.043***
	(0.016)	(0.013)
topic: incentive wage	−0.054***	−0.053***
	(0.013)	(0.011)
topic: university	0.064***	−0.038**
	(0.020)	(0.017)
Constant	0.192***	0.153***
	(0.016)	(0.013)
Observations	95	95
R^2	0.288	0.232
Adjusted R^2	0.257	0.198
Residual Std. Error	0.053 (df = 90)	0.045 (df = 90)
F Statistic	9.121*** (df = 4; 90)	6.794*** (df = 4; 90)
Note:		*p<0.1; **p<0.05; ***p<0.01

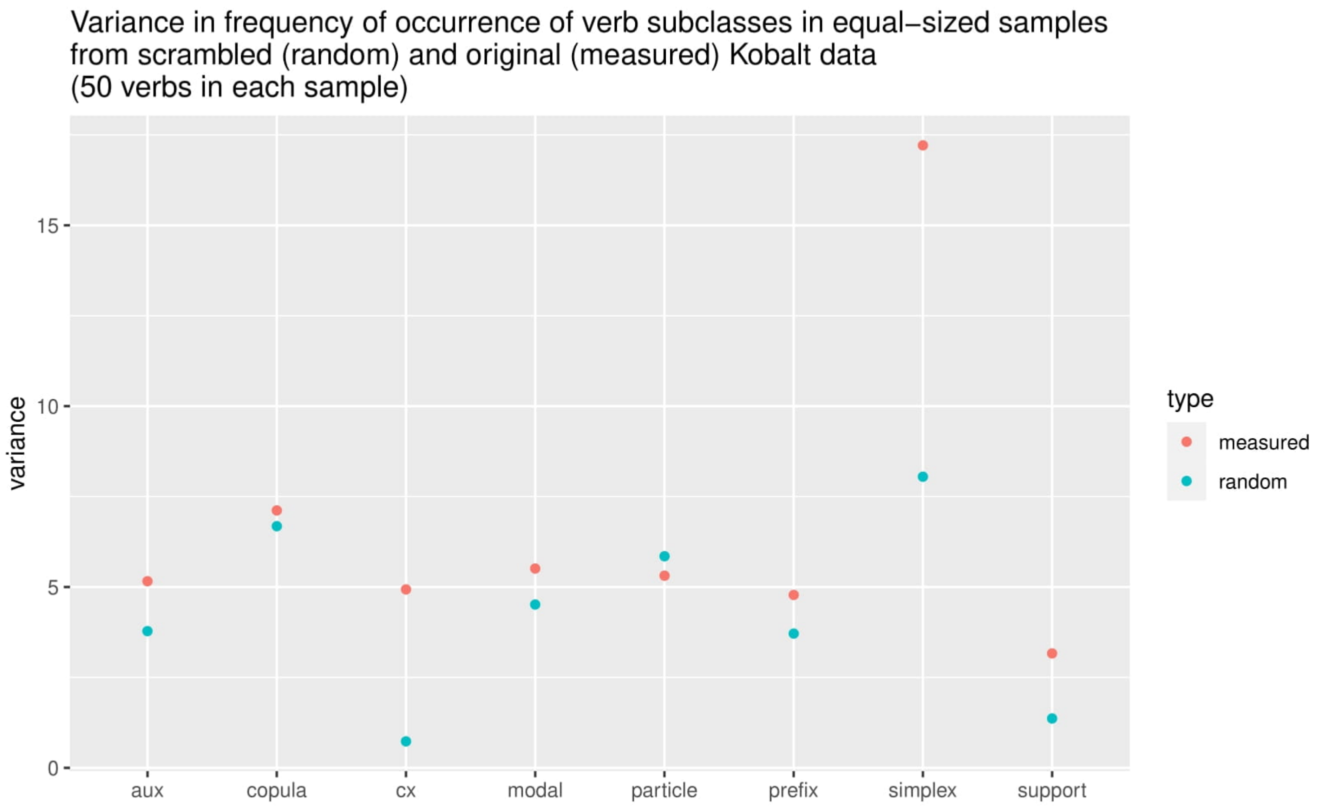

Figure S2. Variance in the frequency of occurrence in cross-corpus randomized and original (text-dependent) Kobalt data.

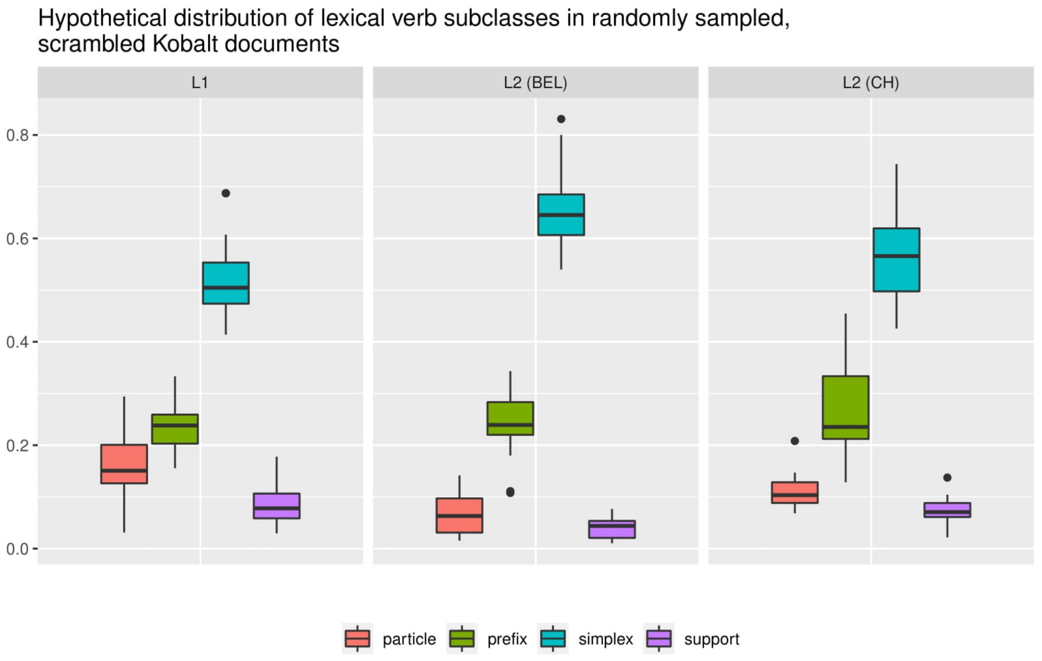

Figure S3. For this plot, we randomly sampled tokens from each subcorpus based on the real distribution of text lengths. For instance, if there were texts of 854, 912, 638 tokens in length, we sampled 854, 912, and 638 tokens from the corpus without replacement. Sampled tokens are not grammatically constrained. They serve to demonstrate the expected variance based on the frequency of occurrence of each category in the respective subcorpus.

Table S3. Continued statistics for proportion of subclasses depending on text length and topic. Significant values for text length dependency are marked in red.

	Dependent variable: proportion of	
	simplex nouns	transpositions
text length	0.0001***	−0.00002
	(0.00002)	(0.00002)
topic: feminism	0.129***	−0.063***
	(0.019)	(0.013)
topic: incentive wage	0.016	−0.039***
	(0.015)	(0.011)
topic: university	−0.105***	−0.049***
	(0.024)	(0.016)
Constant	0.109***	0.107***
	(0.019)	(0.013)
Observations	95	94
R^2	0.523	0.283
Adjusted R^2	0.502	0.251
Residual Std. Error	0.063 (df = 90)	0.043 (df = 89)
F Statistic	24.647*** (df = 4; 90)	8.788*** (df = 4; 89)
Note:	*p<0.1; **p<0.05; ***p<0.01	
